# CENP-C-targeted PLK-1 regulates kinetochore function in *C. elegans* embryos

**DOI:** 10.1242/jcs.262327

**Published:** 2024-11-28

**Authors:** Laura Bel Borja, Samuel J. P. Taylor, Flavie Soubigou, Federico Pelisch

**Affiliations:** Molecular, Cellular and Developmental Biology, School of Life Sciences, University of Dundee, Dundee DD1 5EH, UK

**Keywords:** CCAN, CENP-C, MIS12, PLK, Kinetochore, Mitosis

## Abstract

Polo-like kinase 1 (PLK-1) is present in centrosomes, the nuclear envelope and kinetochores and plays a significant role in meiosis and mitosis. PLK-1 depletion or inhibition has severe consequences for spindle assembly, spindle assembly checkpoint (SAC) activation, chromosome segregation and cytokinesis. BUB-1 targets PLK-1 to the outer kinetochore and, in mammals, the inner kinetochore PLK1 targeting is mediated by the constitutive centromere associated network (CCAN). BUB-1-targeted PLK-1 plays a key role in SAC activation and has a SAC-independent role through targeting CDC-20. In contrast, whether there is a specific, non-redundant role for inner kinetochore targeted PLK-1 is unknown. Here, we used the *Caenorhabditis elegans* embryo to study the role of inner kinetochore PLK-1. We found that CENP-C, the sole CCAN component in *C. elegans* and other species, targets PLK-1 to the inner kinetochore during prometaphase and metaphase. Disruption of the CENP-C–PLK-1 interaction leads to an imbalance in kinetochore components and a defect in chromosome congression, without affecting CDC-20 recruitment. These findings indicate that PLK-1 kinetochore recruitment by CENP-C has at least partially distinct functions from outer kinetochore PLK-1, providing a platform for a better understanding of the different roles played by PLK-1 during mitosis.

## Teaser

**Highlighted Article:** In *C. elegans* embryos, PLK-1 targeted to the inner kinetochore by CENP-C has different functions from when recruited to the outer kinetochore by BUB-1.

## INTRODUCTION

Polo-like kinases (PLKs) are a family of serine/threonine kinases that play essential roles during the cell cycle (Zitouni et al., 2014). PLKs can localize in the centrosome, nuclear envelope, and kinetochore. Targeting to the different locations is dependent on the PLK C-terminal polo-binding domain (PBD) binding to short, phosphorylated motifs at S-S/T-X, where the central S/T is the phosphorylated residue (Elia et al., 2003a,b). In many cases, X is a proline residue, and the STP motif constitutes a CDK-dependent PLK-binding motif.

Within the kinetochore, PLK1 (PLK-1 in *Caenorhabditis elegans*, hereafter referred to as PLK-1 regardless of species) plays important roles in regulating the spindle assembly checkpoint (SAC) and kinetochore–microtubule attachments (Elowe et al., 2007, 2010; von Schubert et al., 2015; Jia et al., 2016; Espeut et al., 2015). Although a plethora of PLK-binding proteins have been described to date, PLK-1 kinetochore targeting is largely dependent on BUB-1 in the outer kinetochore and the constitutive centromere**-**associated network (CCAN) in the inner kinetochore (Singh et al., 2021). In contrast to the mechanistic understanding of kinetochore targeting, little is known about the roles and substrates in the different kinetochore subpopulations. Recruited by BUB-1, outer kinetochore PLK-1 regulates CDC-20 phosphorylation and regulates the spindle assembly checkpoint (Taylor et al., 2023) and mitosis timing (Houston et al., 2023). In contrast, little is known about the role of inner-kinetochore PLK1. Although it is known that CENP-U is the CCAN component targeting PLK1 to the inner kinetochore in mammalian cells, we have recently described that during *C. elegans* oocyte meiosis, the sole *C. elegans* CCAN component, the CENP-C-like protein HCP-4 (hereafter designated CENP-C), can target PLK-1 to different chromosome domains from BUB-1. Interestingly, the different PLK-1 populations appear to play somewhat different roles. *C. elegans* provides an excellent model system to study the mitotic divisions, and we therefore decided to address the outstanding question on the role of inner kinetochore targeted PLK-1 using the first embryonic division.

## RESULTS AND DISCUSSION

### Inner kinetochore recruitment of PLK-1 by CENP-C

**Fig. 1.**
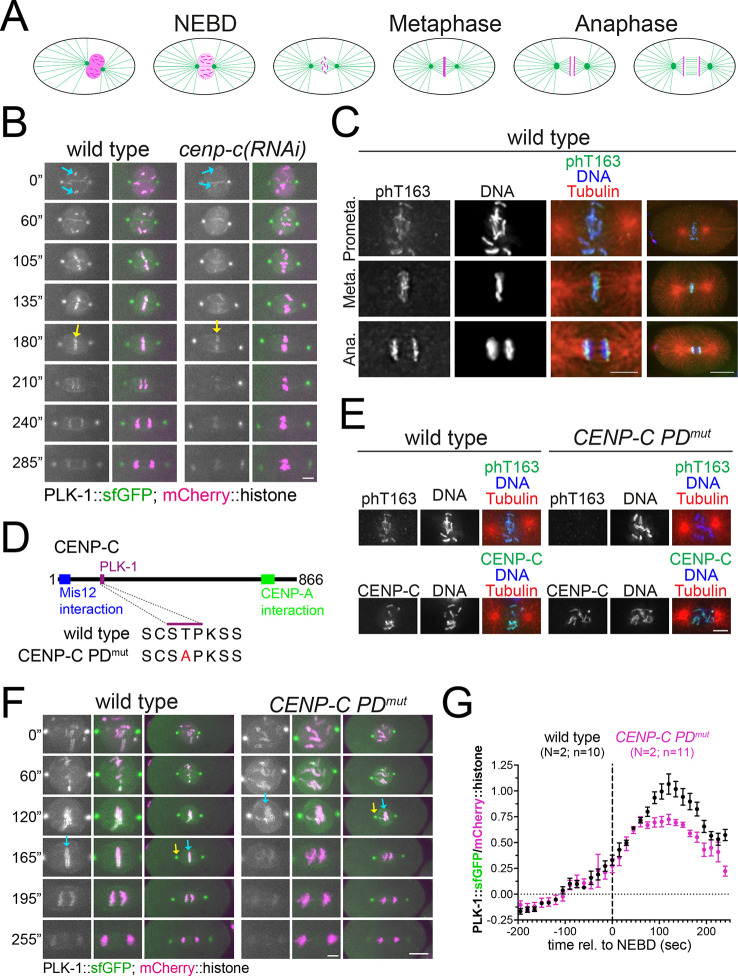
**CENP-C is required for PLK-1 kinetochore localization.** (A) Schematic of the different stages of the first mitotic division in *C. elegans* embryos. (B) Fluorescently labeled PLK-1 was followed throughout mitosis in wild-type and CENP-C-depleted embryos [*cenp-c(RNAi)*]. Prometaphase and metaphase chromosomes are highlighted by cyan and yellow arrows, respectively. Note that images in wild type and CENP-C PD^mut^ embryos have been matched for mitotic stage. Images are representative of wild-type, *N*=2; *n*=14; CENP-C PD^mut^, *N*=2; *n*=11. Scale bars: 5 µm. (C) Phosphorylation of T163 (phT163) within the PD motif in CENP-C was detected in fixed wild-type embryos using a phospho-specific antibody. Images from prometaphase (Prometa.), Metaphase (Meta.), and Anaphase (Ana.) are shown. Images are representative of three embryos and the experiment was repeated three times. Scale bars: 4 µm (magnified panels); 10 µm (full-embryo images on left). (D) Schematic indicating the PD motif characterized previously (Taylor et al., 2023) along with its mutant sequence (CENP-C PD^mut^), and the putative Mis12 and CENP-A interaction regions. (E) Top panelsm phosphorylation of T163 in CENP-C was detected using a phospho-specific antibody in fixed wild-type and T163A CENP-C mutant (*CENP-C PD^mut^*) embryos; note that wild-type images are reproduced from wild-type prometaphase (top) panel in C. Bottom panels, wild-type and CENP-C PD^mut^ embryos were stained for CENP-C. Images are representative of three embryos and the experiment was repeated three times. Scale bar: 4 µm. (F) Fluorescently labeled PLK-1 was followed throughout mitosis in wild-type and polo-docking mutant CENP-C (*CENP-C PD^mut^*). Yellow arrows point to the centrosome and cyan arrows to metaphase chromosomes. Note that images in wild-type and CENP-C PD^mut^ embryos have been matched for mitotic stage. Scale bars: 3 µm (magnified panels); 10 µm (full-embryo images). (G) Chromosomal PLK-1 levels were quantified and normalized to the chromosome signal. Values shown represent the mean±s.e.m. Where indicated, N denotes the number of experiments and n, is the number of embryos analyzed.

We have recently identified *C. elegans* CENP-C as a PLK-1 receptor (Taylor et al., 2023). We decided to assess whether CENP-C-targeted PLK-1 plays a role during mitosis and, if so, whether this differs from that of BUB-1-targeted PLK-1 (Houston et al., 2023). Key stages of the first mitotic division of the *C. elegans* embryo are highlighted in Fig. 1A for reference. We used endogenously sfGFP-tagged PLK-1 (Martino et al., 2017) to study its kinetochore recruitment and found a clear reduction in chromosomal PLK-1 levels after CENP-C depletion [*cenp-c(RNAi)*] at all mitosis stages (Fig. 1B; Movies 1, 2). T163 phosphorylation within the polo docking (PD) motif in CENP-C was detected in fixed embryos using a phospho-specific antibody (Fig. 1C) (Taylor et al., 2023) and, as expected this signal was lost in the PD mutant (*cenp-c^T163A^*, hereafter referred to as CENP-C PD^mut^) (Fig. 1D,E). Given that CENP-C is required for kinetochore assembly, lower chromosomal PLK-1 levels could be the trivial consequence of depleting BUB-1 from the kinetochore. We therefore used the CENP-C PD^mut^, which cannot target PLK-1 (Taylor et al., 2023), but where CENP-C is still present on chromatin in embryos (Fig. 1E, lower panels). Using live embryo imaging, we found that kinetochore PLK-1 levels were significantly reduced in the CENP-C PD^mut^ embryos (Fig. 1F, blue arrows; Movies 3, 4). This reduction became apparent at ∼80 s after nuclear envelope breakdown (NEBD; Fig. 1F,G; Movies 3, 4) and showed the maximal difference around metaphase, where PLK-1 kinetochore levels in CENP-C PD^mut^ were down to ∼65% (Fig. 1G). Importantly, this reduction was also observed in immunofluorescence images of fixed samples using a specific anti-PLK-1 antibody (Fig. S1A). Abolishing PLK-1 binding to CENP-C did not affect PLK-1 at centrosomes (Fig. 1F, yellow arrows) and did not significantly change BUB-1 levels (Fig. S1B,C). These results suggest that CENP-C represents a parallel pathway for PLK-1 recruitment during mitosis, much like CENP-U in mammalian cells (Singh et al., 2021; Kang et al., 2011, 2006).

### PLK-1 targeting by CENP-C is not involved in CDC-20 kinetochore recruitment

**Fig. 2.**
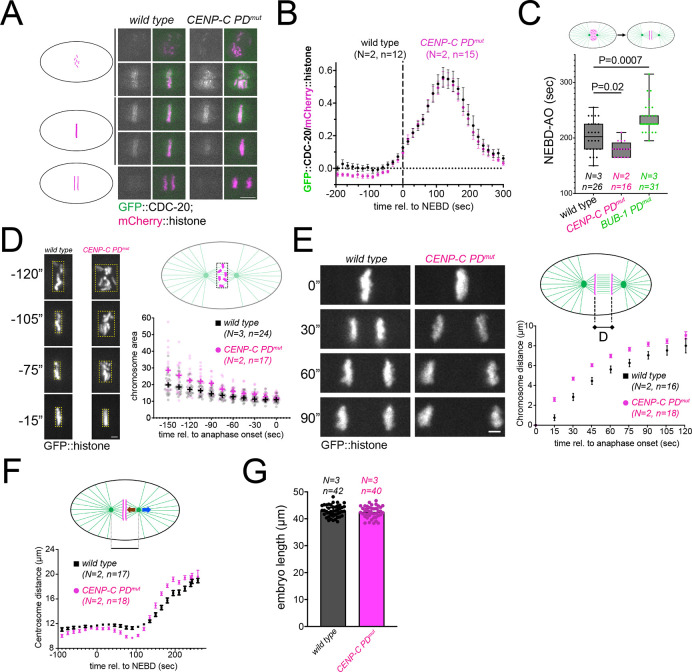
**CENP-C–PLK-1 binding is not necessary for CDC-20 kinetochore recruitment.** (A) Fluorescently labeled CDC-20 was followed throughout mitosis in wild-type and polo-docking mutant CENP-C (*CENP-C PD^mut^*) embryos. Note that images in wild-type and CENP-C PD^mut^ embryos have been matched for mitotic stage. Scale bar: 5 µm. (B) Chromosomal CDC-20 levels were quantified and normalized to the chromosome signal. Values shown represent the mean±s.e.m. (C) The time elapsed between NEBD and anaphase onset was measured. The box represents the 25–75th percentiles, and the median is indicated. The whiskers show the range. Circles denote the individual measurements. We performed Kruskal–Wallis test and Dunn's multiple comparisons test, and the *P* values are reported in the graph. (D) Chromosome congression was assessed by bounding box analysis and the graph shows the chromosome (bounding box) area over time (″ representing seconds). Circles represent the individual measurements and the plus signs are the median for each time point. Scale bar: 2 µm. (E) Chromosome distance was measured and the mean±s.e.m. is represented. Scale bar: 2 µm. (F) Centrosome-to-centrosome distance was measured and the mean±s.e.m. is represented. (G) Embryo length was measured in wild-type and *CENP-C PD^mut^* embryos. Results are median±95% c.i. Where indicated, *N* denotes the number of experiments and *n*, is the number of embryos analyzed.

Given that BUB-1-targeted PLK-1 is necessary for CDC-20 kinetochore localization (Houston et al., 2023), we assessed whether CENP-C-targeted PLK-1 is also involved in CDC-20 kinetochore targeting. We followed CDC-20 kinetochore localization throughout mitosis and found that mutation of the CENP-C PD motif has no impact on CDC-20 kinetochore localization (Fig. 2A,B; Movies 5, 6). To further compare the impact of disrupting CENP-C–PLK-1 binding on BUB-1–PLK-1 binding, we assessed mitotic timing, as measured by the time from NEBD to anaphase onset. Whereas BUB-1 PD^mut^ embryos displayed a longer mitosis duration, as described previously (Houston et al., 2023), CENP-C PD^mut^ embryos displayed a slightly reduced mitotic duration (Fig. 2C). These results indicate that inner kinetochore targeting of PLK-1 by CENP-C regulates different mitotic events from BUB-1-targeted PLK-1.

### PLK-1 targeting by CENP-C regulates chromosome congression and kinetochore-microtubule interactions

What mitotic events are regulated by CENP-C-targeted PLK-1? To answer this question, we monitored chromosome congression, chromosome segregation and kinetochore–microtubule interactions. To assess chromosome congression, we used a ‘bounding box’ method whereby we measured the area of the minimal box that surrounds all chromosomes throughout mitosis (Fig. 2D; Movies 7, 8). This analysis revealed that disrupting PLK-1 targeting by CENP-C leads to significant chromosome congression defects (Fig. 2D; Movies 7, 8). Immediately prior to anaphase onset, chromosomes do manage to congress, and the difference between wild-type and CENP-C PD^mut^ embryos becomes minimal. Our next focus was chromosome segregation during anaphase, and we measured the distance between segregating chromosomes as anaphase progresses (Fig. 2E; Movies 9, 10). Abolishing PLK-1 targeted BUB-1 has no appreciable effect on chromosome segregation (Houston et al., 2023). In contrast, inhibiting PLK-1 binding to CENP-C led to faster chromosome separation (Fig. 2E; Movies 9, 10). Interestingly, increased chromosome separation in the *C. elegans* first mitotic division is characteristic of kinetochore defects (Maton et al., 2015). Prompted by this result, we decided to assess kinetochore–microtubule interactions. We measured the distance between centrosomes during mitosis, whose net movement results from the balance between astral and kinetochore microtubules (Fig. 2F, blue and brown arrows on the embryo picture). Hence, the kinetics of spindle pole separation is used to infer the strength of kinetochore–microtubule attachments (Cheerambathur et al., 2017; Desai et al., 2003; Oegema et al., 2001). Interestingly, we noticed two significant differences between wild-type and CENP-C PD^mut^ embryos. Spindle poles were significantly closer between 45 and 120 s after NEBD (before sister chromatid separation) in the CENP-C PD^mut^ (Fig. 2F). This suggests that forces exerted by kinetochore microtubules are pulling stronger than the astral microtubules during this time window. The second difference was apparent during anaphase, when sister chromatids are segregating, and centrosomes begin to separate. The rate of centrosome separation was significantly higher in CENP-C PD^mut^ embryos (Fig. 2F). As opposed to the pre-anaphase imbalance in forces, this would suggest that forces exerted by astral microtubules are not balanced by kinetochore microtubules. Importantly, the defects in congression and segregation in the CENP-C PD^mut^ are not the result of differences in embryo length (Fig. 2G).

Altogether, the phenotypes observed in CENP-C PD^mut^ embryos suggest that CENP-C-bound PLK-1 has a role in regulating kinetochore function.

### Inner kinetochore targeted PLK-1 regulates outer kinetochore assembly

**Fig. 3.**
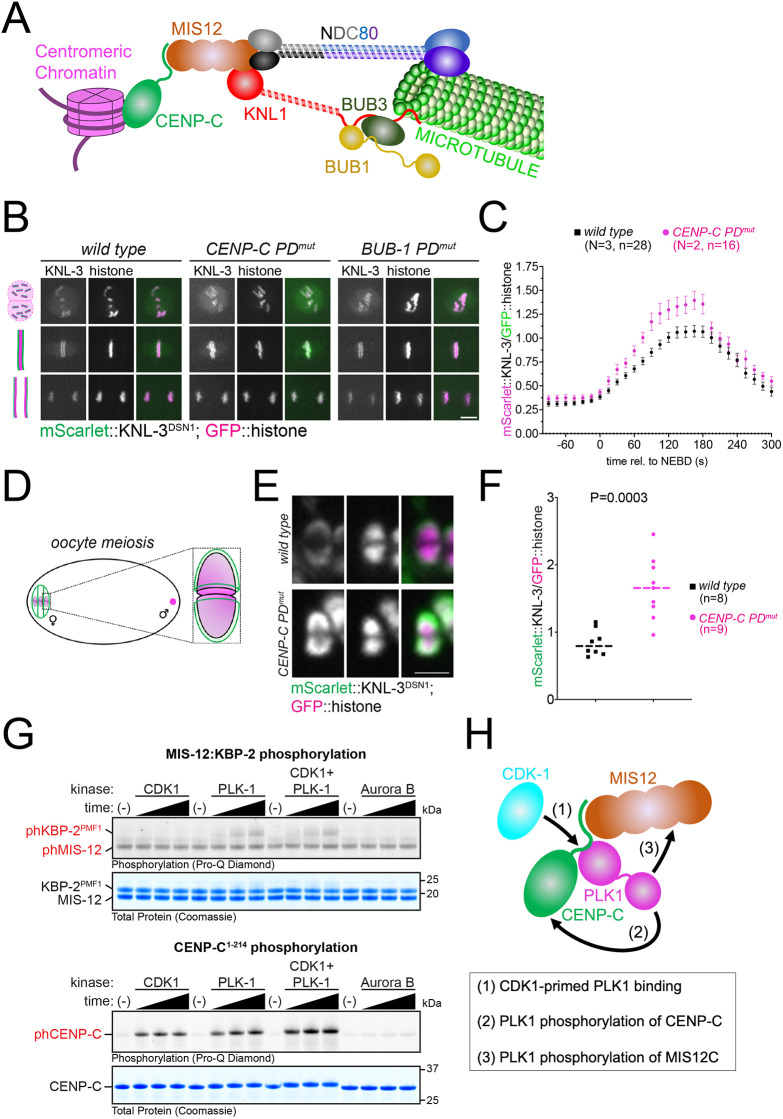
**KNL-3^DSN1^ kinetochore localization in the CENP-C PD^mut^.** (A) Schematic depicting how CENP-C connects the centromeric chromatin with the outer kinetochore, consisting of the KMN network (KNL-1–Mis12 complex and NDC80 complex) and the BUB complex (BUB-1 and BUB-3). The NDC-80 complex provides the major point of contact with microtubules. (B) mScarlet-tagged endogenous KNL-3 was followed throughout mitosis in wild-type and polo-docking mutant CENP-C (*CENP-C PD^mut^*) embryos. Scale bar: 5 µm. (C) Chromosomal KNL-3 levels were quantified and normalized to the chromosome signal. Values shown represent the mean±s.e.m. *N* denotes the number of experiments and *n*, the number of embryos analyzed. (D) Schematic of an oocyte with a zoomed schematic image of a bivalent. Chromosomes are shown in magenta and kinetochores in green. (E) mScarlet-tagged endogenous KNL-3 was imaged during oocyte meiosis and a single image corresponding to prometaphase I is shown for wild-type and *CENP-C PD^mut^* embryos. Scale bar: 2 µm. (F) Chromosomal KNL-3 levels were quantified and normalized to the chromosome (histone) signal. Individual values are shown, and the dashed line represents the median. ‘*n*’ denotes the number of embryos analyzed. Wild-type and CENP-C PD^mut^ embryos were compared using a non-parametric test (Mann–Whitney) and the *P*-value is shown. (G) Kinase assays were performed with the indicated protein kinase and CENP-C or the KBP-2:MIS-12 dimer. Time points analyzed were 15, 30 and 45 min (black triangle). Image shown is representative of three experimental repeats. (H) Summary of the putative sequence of events leading to KBP-2 phosphorylation by PLK-1.

To gain more insight into how kinetochore function is affected in CENP-C PD^mut^ embryos, we followed the dynamics of two outer kinetochore complexes: MIS12, which interacts directly with CENP-C in the inner kinetochore, and NDC80, which is present further away from centromeric chromatin and is the main point of microtubule interaction (Fig. 3A). We imaged endogenously tagged MIS-12 complex subunit KNL-3 (the homolog of mammalian DSN1; hereafter denoted KNL-3^DSN1^) during mitosis and found that loading onto chromatin started at a similar time at NEBD in both wild type and CENP-C PD^mut^ (Fig. 3B,C; Movies 11–13). Immediately after NEBD, the rate of KNL-3^DSN1^ loading onto kinetochores was faster in the CENP-C PD^mut^ embryos (Fig. 3B,C; Movies 11–13), also reaching a higher maximum level compared to wild-type embryos (30% higher at 165 s post-NEBD). KNL-3^DSN1^ dissociated from chromatin during anaphase with similar dynamics in both wild-type and CENP-C PD^mut^ embryos (Fig. 3B,C; Movies 11–13). CENP-C-targeted PLK-1 also regulates the MIS12 complex during female meiosis: CENP-C PD^mut^ oocytes had higher levels of kinetochore-associated KNL-3^DSN1^ (Fig. 3D,E), demonstrating that kinetochore protein loading during both meiosis and mitosis is controlled by CENP-C-targeted PLK-1. A similar increase in kinetochore loading was observed for the NDC80 complex component NDC-80 during mitosis and meiosis (Fig. S2A­–D).

These results suggest that inner kinetochore PLK-1 limits the amount of outer kinetochore proteins loaded onto chromatin between NEBD and anaphase onset and could explain the greater force exerted by kinetochores in the CENP-C PD^mut^ in Fig. 2F.

In mammals and *Drosophila*, interaction with CENP-C is mainly mediated by the MIS12–PMF1 dimer (Petrovic et al., 2016; Richter et al., 2016). We purified a recombinant N-terminal fragment of *C. elegans* CENP-C (Taylor et al., 2023) and the *C. elegans* homolog of PMF1, KBP-2 (denoted KBP-2^PMF1^) to make a KBP-2^PMF1^–MIS-12 dimer (Fig. S3) and analyzed their phosphorylation status after incubation with CDK1 and PLK-1 (alone or in combination, Fig. 3G). We found that whereas CDK1 phosphorylated CENP-C, as we described before (Taylor et al., 2023), and not the KBP-2^PMF1^–MIS-12 dimer; PLK-1 phosphorylated both CENP-C and the KBP-2^PMF1^–MIS-12 dimer (Fig. 3G). Interestingly, whereas CDK1 and PLK-1 together further enhanced CENP-C phosphorylation, no such increase was observed for the KBP-2^PMF1^–MIS-12 dimer compared to PLK-1 on its own (Fig. 3G). The MIS-12 complex KNL-3 displayed a similar behavior to the KBP-2^PMF1^–MIS-12 dimer (Fig. S3D). *In vitro* reactions were analyzed by mass spectrometry and all phospho-site data can be found in Table S1. We also tested Aurora B given that it phosphorylates KNL-3^DSN1^ within the MIS12 complex (Welburn et al., 2010) but it had no impact on CENP-C or KBP-2^PMF1^–MIS-12 phosphorylation levels (Fig. 3G). A plausible model stemming from these results is that CDK1 phosphorylates CENP-C in the inner kinetochore driving PLK-1 recruitment. PLK-1 would then phosphorylate CENP-C and KBP-2^PMF1^ (among other putative substrates) to regulate kinetochore function (Fig. 3H). Further work will be required to characterize the landscape of PLK-1 substrates.

### Disruption of inner and outer kinetochore PLK-1 leads to severe defects in mitotic chromosome segregation

**Fig. 4.**
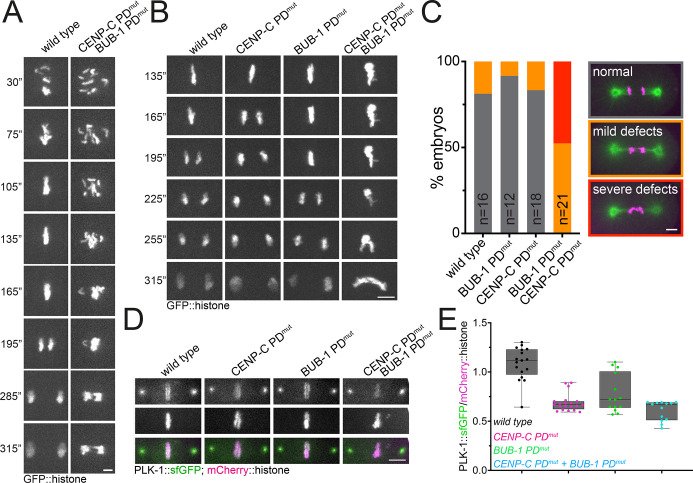
**Combined abrogation of binding of PLK-1 to both CENP-C and BUB-1 leads to severe mitotic defects.** (A) GFP-tagged histone was followed throughout mitosis in wild-type and polo docking *CENP-C PD^mut^* and *BUB-1 PD^mut^* embryos. Times are relative to NEBD (0″, where ″ is seconds). Images are representative of wild-type, *N*=2; *n*=14; CENP-C PD^mut^ BUB-1 PD^mut^, *N*=2; *n*=17. Scale bar: 3 µm. (B) GFP-tagged histone was followed throughout mitosis in wild-type and the *CENP-C PD^mut^* and *BUB-1 PD^mut^* double-mutant embryos. Times are relative to NEBD (0″). Images are representative of wild-type *N*=2; *n*=14; CENP-C PD^mut^, *N*=2; *n*=19; BUB-1 PD^mut^, *N*=2; *n*=14; CENP-C PD^mut^ BUB-1 PD^mut^, *N*=2; *n*=12. Scale bar: 5 µm. (C) Chromosome segregation defects were arbitrarily divided into mild and severe (see Materials and Methods) and the percentage of embryos displaying any phenotype for each condition was quantified. *n* denotes the number of embryos analyzed for each condition. Scale bar: 5 µm. (D) Chromosomal PLK-1 levels were analyzed in wild-type, CENP-C PD^mut^, BUB-1 PD^mut^ embryos and double *CENP-C PD^mut^* and *BUB-1 PD^mut^* double-mutant, and representative images are shown. (E) Kinetochore PLK-1 levels were quantified and normalized to the chromosome (mCherry::histone) signal. The box represents the 25–75th percentiles, and the median is indicated. The whiskers show the range. Individual points (single embryos) are shown. Quantitation corresponds to 135 s post-NEBD.

Having established that inner kinetochore targeted PLK-1 plays different roles to outer kinetochore PLK-1 during mitosis, we decided to assess the consequences of inhibiting PLK-1 recruitment by both BUB-1 and CENP-C. Chromosome alignment and segregation were severely affected by the combined mutation of PLK-1 docking sites (Fig. 4A; Movies 14, 15). We decided to focus our further analysis on chromosome segregation (Fig. 4B,C; Movies 16–19) and found that 100% of CENP-C PD^mut^ and BUB-1 PD^mut^ double-mutant embryos displayed abnormal segregation, which included mild (52%) and severe (48%) defects (Fig. 4C). Interestingly, although the combined CENP-C and BUB-1 PD mutants displayed lower PLK-1 levels than BUB-1 PD^mut^ (Fig. 4D,E; *P*=0.025 Mann–Whitney test), the difference was not significant compared to that seen for the CENP-C PD^mut^ alone (Fig. 4D,E; *P*=0.25 Mann–Whitney test). These results show that the severity of the phenotypes does not correlate with the quantity of PLK-1 and therefore suggest that a qualitative difference (i.e. substrates) might explain the phenotypes. It should be noted that some PLK-1 is still detectable in kinetochores, which could be due to (1) the small amount of endogenous BUB-1 remaining in the BUB-1^T527A^ mutant condition (see Materials and Methods) and/or (2) the existence of another PLK-1 centromere/kinetochore receptor. In summary, joint disruption of the main inner and outer kinetochore platforms for PLK-1 recruitment leads to severe mitotic defects, reminiscent of the joint depletion of CENP-U and BUB1 in mammalian cells (Chen et al., 2021).

We have provided evidence supporting a specific role for inner kinetochore-bound PLK-1 during mitotic chromosome segregation. Interestingly, CENP-C-bound PLK-1 appears to play a more important role than CENP-U in mammals given that the abrogation of CENP-C–PLK-1 binding leads to kinetochore–microtubule attachment defects as well as chromosome congression defects. In mammalian cells, these effects are seen mainly when CENP-U–PLK-1 binding is abrogated in cells not expressing BUB1 (Chen et al., 2021). Our experiments using point mutants instead of depletion to abolish PLK1 binding to BUB-1 and CENP-C show that there is still PLK-1 remaining on kinetochores, suggesting the existence of other pathway(s) targeting PLK-1 to kinetochores. However, this remaining kinetochore PLK-1 population is not sufficient to sustain proper chromosome segregation. We also provide additional insight by describing that MIS12 and NDC80 complexes increase at kinetochores in the absence of inner kinetochore-bound PLK-1. It will be important to identify inner and outer kinetochore PLK-1 substrates to understand the mechanisms underlying the different functions of PLK-1. Additionally, we are also focusing on identifying additional PLK-1 receptors.

## MATERIALS AND METHODS

### *C. elegans* strains and RNAi

Strains used in this study were maintained at 20°C unless indicated otherwise. For a complete list of strains, please refer to Table S2.

For RNAi-mediated depletions, the targeting sequence for *bub-1* was 2353–2935 and for *hcp-4*, 967–2128, both from the first ATG codon. For double depletion, both sequences were cloned in the same vector. All sequences were inserted into L4440 using the NEBuilder HiFi DNA Assembly Master Mix (New England Biolabs) and transformed into DH5α bacteria. The purified plasmids were then transformed into HT115(DE3) bacteria (Timmons et al., 2001). RNAi clones were picked and grown overnight at 37°C in lysogeny broth (LB) with 100 μg/ml ampicillin (Formedium). Saturated cultures were diluted 1:100 and allowed to grow until reaching an optical density at 600 nm (OD600) of 0.8–1. Isopropyl-β-D-thiogalactopyranoside (IPTG) was added to a final concentration of 1 mM, and cultures were incubated for 1 h at 37°C. Bacteria were then seeded onto nematode growth medium (NGM) plates made with agarose and 1 mM IPTG and allowed to dry. L4 stage worms were then plated on RNAi plates and maintained at 20°C.

### Live imaging of embryos

Embryos were dissected and mounted in 5 µl of L-15 blastomere culture medium [0.5 mg/ml inulin (Sigma), 25 mM HEPES, pH 7.5 in 60% Leibowitz L-15 medium (#11415049, Thermo Fisher Scientific) and 20% heat-inactivated FBS (#A5670801, Thermo Fisher Scientific)] on 24×40 mm #1.5 coverslips. After dissection and isolation of early embryos, a circle of Vaseline was laid around the sample, and a custom-made 24×40 mm plastic holder (with a centered window) was placed on top. The sample was imaged immediately using the 488 nm and 561 nm laser lines. Live imaging was done using a CFI Plan Apochromat Lambda 60× NA 1.4 oil objective mounted on a microscope (Nikon Eclipse Ti) equipped with a Prime 95B 22 mm camera (Photometrics) and a spinning-disk head (CSU-X1; Yokogawa Electric Corporation). Acquisition parameters were controlled with NIS software (Nikon). For all live imaging experiments, partial projections are presented. All files were stored, classified, and managed using OMERO (Allan et al., 2012). Figures were prepared using OMERO.figure and assembled using Adobe Illustrator. Representative movies shown in the supplementary material (Movies 1–19) were assembled using Fiji/ImageJ (Schindelin et al., 2012) with custom-made macros (available upon request).

In Fig. 3B, the strains used were FGP739, FGP740 and FGP741, all in the presence of *bub-1(RNAi)* to deplete endogenous BUB-1. In Fig. 3E, the strains used were FGP722 and FGP728. In Fig. S2A, mitotic analysis was performed using strains FGP803, FGP804 and FGP805, all in the presence of *bub-1(RNAi)* to deplete endogenous BUB-1. In Fig. S2C (meiotic analysis), FGP372 and FGP729 strains were used.

### Kinase assays

Reactions were carried out using 55 nM human CDK1–cyclinB (Thermo Fisher Scientific), 75 nM *C. elegans* PLK-1 (produced in house) or 200 nM human Aurora B (MRC PPU Reagents and Services, #DU1773) in 50 mM Tris-HCl pH 7.5, 1 mM ATP, 10 mM MgCl_2_, 0.5 M TCEP and 0.1 mM EDTA. Substrates were used at ∼16 µM (CENP-C; produced in-house) or ∼10 µM (KBP-2–MIS-12 dimer; produced in-house). Reactions were conducted at 30°C for the indicated time points. Samples were taken and added to an equal volume of 2× LDS buffer (Thermo Fisher Scientific) and incubated at 70°C for 15 min or 95°C for 5 min before loading onto SDS-PAGE gels.

### Immunofluorescence

Worms were placed on 4 μl of M9 worm buffer in a poly-D-lysine (Sigma, P1024)-coated slide and a 24×24-cm coverslip was gently laid on top. Worms were dissected to release the embryos; slides were placed on a metal block on dry ice for >10 min. The coverslip was then flicked off with a scalpel blade, and the samples were fixed in methanol at 20°C for 30 min. Primary antibodies were: anti-tubulin (1:400, clone DM1A, Merck); anti-PLK-1 (1:400, Budirahardja and Gönczy, 2008); anti-HCP-4 (1:2000, Oegema et al., 2001); and anti-phospho-Thr 163 HCP-4 (1:2000, Taylor et al., 2023). Secondary antibodies were goat anti-rabbit-IgG and goat anti-mouse-IgG conjugated to Alexa Fluor™ 488 and Alexa Fluor™ 594, respectively (1:1,000, Thermo Fisher Scientific). Embryos were mounted in ProLong Diamond antifade mountant (Thermo Fisher Scientific) with DAPI.

### Cloning and expression of the KBP-2^PMF1^–MIS-12 dimer

Codon-optimized 6×His-TEV-KBP-2 and MIS-12a^1-163^ were cloned into the pET Duet-1 vector (Novagen) using a single-step Gibson Assembly procedure (NEBuilder HiFi DNA Assembly, NEB) using the NcoI and XhoI restriction sites to generate plasmid fgp543. Bacterial cultures at OD_600_ of 0.6–0.8 were induced with 100 µM IPTG for 16–18 h at 20°C. Proteins were purified using Co-NTA resin (MRC PPU Reagents and Services) and subjected to TEV cleavage [TEV protease (produced in-house) was added at a mass ratio of 1:100 (substrate to TEV) and incubated overnight at 4°C]. Co-NTA was used to remove the TEV and the tag, and proteins were loaded on a Superdex 75 16/600 column (Cytiva) for size-exclusion chromatography.

### Cloning and expression of the KNL-3

In an attempt to purify the KNL-3^DSN1^–KBP-1 putative dimer, we cloned codon-optimized Strep-tagII-KNL-3^160–343^ and KBP-1^18–149^ into the pACYC Duet-1 vector (Novagen) using a single-step Gibson Assembly procedure (NEBuilder HiFi DNA Assembly, NEB) using the NcoI and XhoI restriction sites to generate plasmid fgp561. Bacterial cultures at an OD_600_ of 0.6–0.8 were induced with 200 µM IPTG for 16–18 h at 20°C. Proteins were purified using StrepTactinXT 4flow resin and loaded on a Superdex 75 10/300 column for size exclusion chromatography. After this procedure, KNL-3 was purified on its own (no interaction with KBP-1).

### Cloning and expression of CENP-C

Cloning and expression of CENP-C^1-214^ were reported previously (Taylor et al., 2023).

### Mass spectrometry phospho-site identification

Liquid chromatography tandem mass spectrometry (LC-MS/MS) experiments to assess *in vitro* phosphorylation were conducted by the Fingerprint Proteomics Facility (University of Dundee). Further details on the LC-MS/MS sample preparation and run can be found in a previous report (Bel Borja et al., 2020). Mascot was used to identify phosphorylated peptides from the mass spectrometry data against a *C. elegans* UniProt protein database. Unless otherwise stated, the following settings were used: trypsin with up to two miscleavages; fixed modifications: carbamidomethyl; variable modifications: phospho(ST)/phospho(Y); monoisotropic experimental mass value; default peptide toleranem (10 ppm); default #^13^C (2); default MS/MS tol. (0.06 Da); default peptide charge (2+ and 3+), instrument: ESI-TRAP. In all analyses, only peptides above the peptide score distribution significance threshold were included in the analysis.

### Protein intensity measurments

A semi-automated Fiji macro (available upon request) was used to measure the levels of the protein of interest (POI). Briefly, a ‘sum slices’ projection was generated for each data point and the signal in the histone channel was taken as a reference. This selection was then transferred to the POI channel and the intensity value was taken. All graphs were generated using Graphpad Prism 10.

### Chromosome congression analysis

The maximum intensity projection in the mCherry::histone channel was used to analyze chromosome area, using the bounding box selection tool in Fiji (Schindelin et al., 2012). Graphs were prepared using Graphpad Prism 10.

### Chromosome segregation defects

Chromosome segregation defects were quantified as belonging two arbitrary categories: (1) ‘mild defects’, when either lagging chromosomes or minimal anaphase bridges were detected and (2) ‘severe defects’, when either severe anaphase bridges or the chromosome masses remained as a single body during anaphase. The researcher doing this analysis was aware of the experimental genotype. The graph was created with Graphpad Prism 10.

## Supplementary Material



10.1242/joces.262327_sup1 Supplementary information
