# Supplementary material for: CENP-C-targeted PLK-1 regulates kinetochore function in C. elegans embryos
Source: J Cell Sci. 2024 Nov 28;137(22):jcs262327. doi: 10.1242/jcs.262327 (PMC11634037; doi:10.1242/jcs.262327)
Supplement: Supplementary information [file joces-137-262327-s1.pdf]

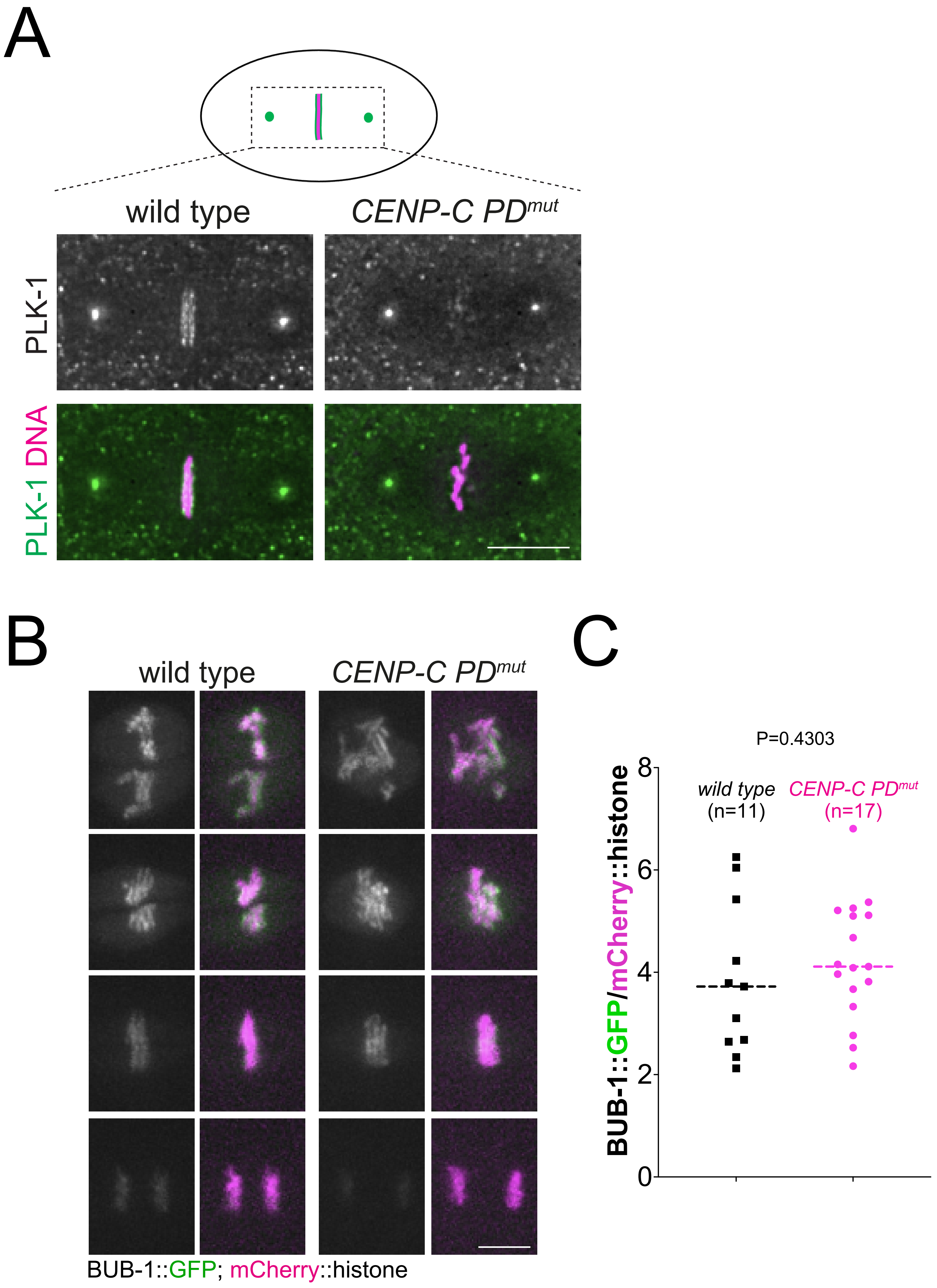


**Fig. S1. CENP-C is required for PLK-1 kinetochore localisation. (A)** Immunofluorescene analysis using a specific anti-PLK-1 antibody was carried out in wild type and CENP-C PD$^{mut}$ embryos at metaphase. Scale bar, 5 µm. **(B)** Fluorescently labelled BUB-1 was followed throughout mitosis in wild type and CENP-C PD$^{mut}$ embryos. Scale bar, 5 µm. **(C)** Chromosomal BUB-1 levels were quantified and normalised to the chromosome (histone) signal. Individual values are shown and thedashed line represents the median. ‘n’ denotes the number of embryos analysed. Wild type and CENP-C PD$^{mut}$ embryos were compared using a non-parametric test (Mann-Whitney) and the P value is shown.

Journal of Cell Science • Supplementary information

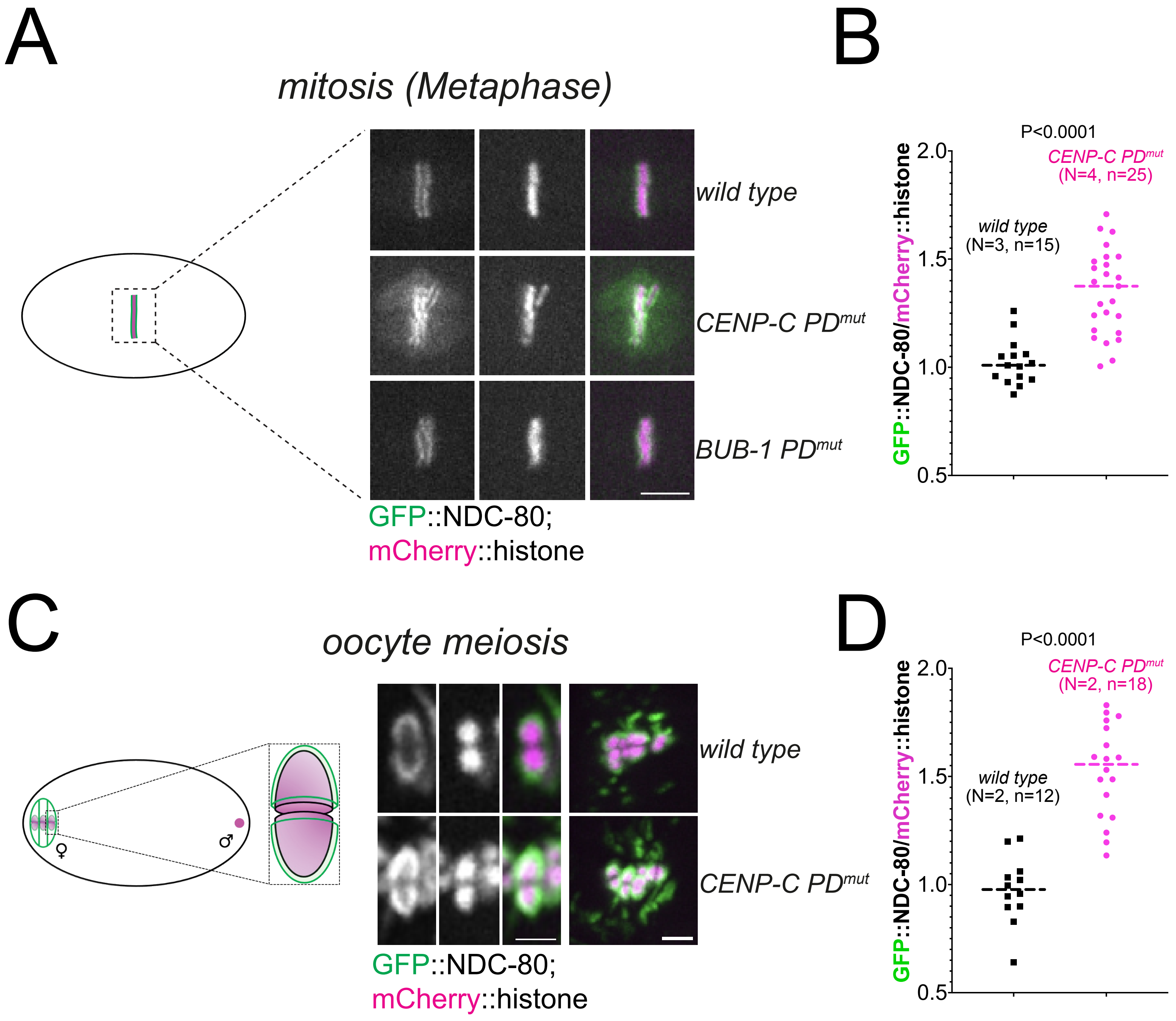


**Fig. S2. NDC-80 kinetochore localisation in the CENP-C PD$^{mut}$.** (A) GFP-tagged endogenous NDC-80 was followed throughout mitosis in wild type, CENP-C PDmut, and BUB-1 PD$^{mut}$. Scale bar, 5 µm. **(B)** Chromosomal NDC-80 levels at metaphase were quantified and normalised to the chromosome (histone) signal. Individual values are shown and thedashed line represents the median. 'N' denotes the number of experiments and 'n' denotes the number of embryos analysed. Wild type and CENP-C PD$^{mut}$ embryos were compared using a non-parametric test (Mann-Whitney) and the P value is shown. **(C)** GFP- tagged endogenous NDC-80 was imaged during oocyte meiosis and a single image corresponding to prometaphase I is shown for wild type and polo docking mutant CENP-C ('*CENP-C PD$^{mut}$*'). Scale bars, 2 µm in the zoomed panel (left) and 3 µm in right panel. **(D)** Chromosomal NDC-80 levels at prometaphase of Meiosis I were quantified and normalised to the chromosome (histone) signal. Individual values are shown and thedashed line represents the median. 'N' denotes the number of experiments and 'n' denotes the number of embryos analysed. Wild type and CENP-C PD$^{mut}$ embryos were compared using a non-parametric test (Mann-Whitney) and the P value is shown.

Journal of Cell Science • Supplementary information

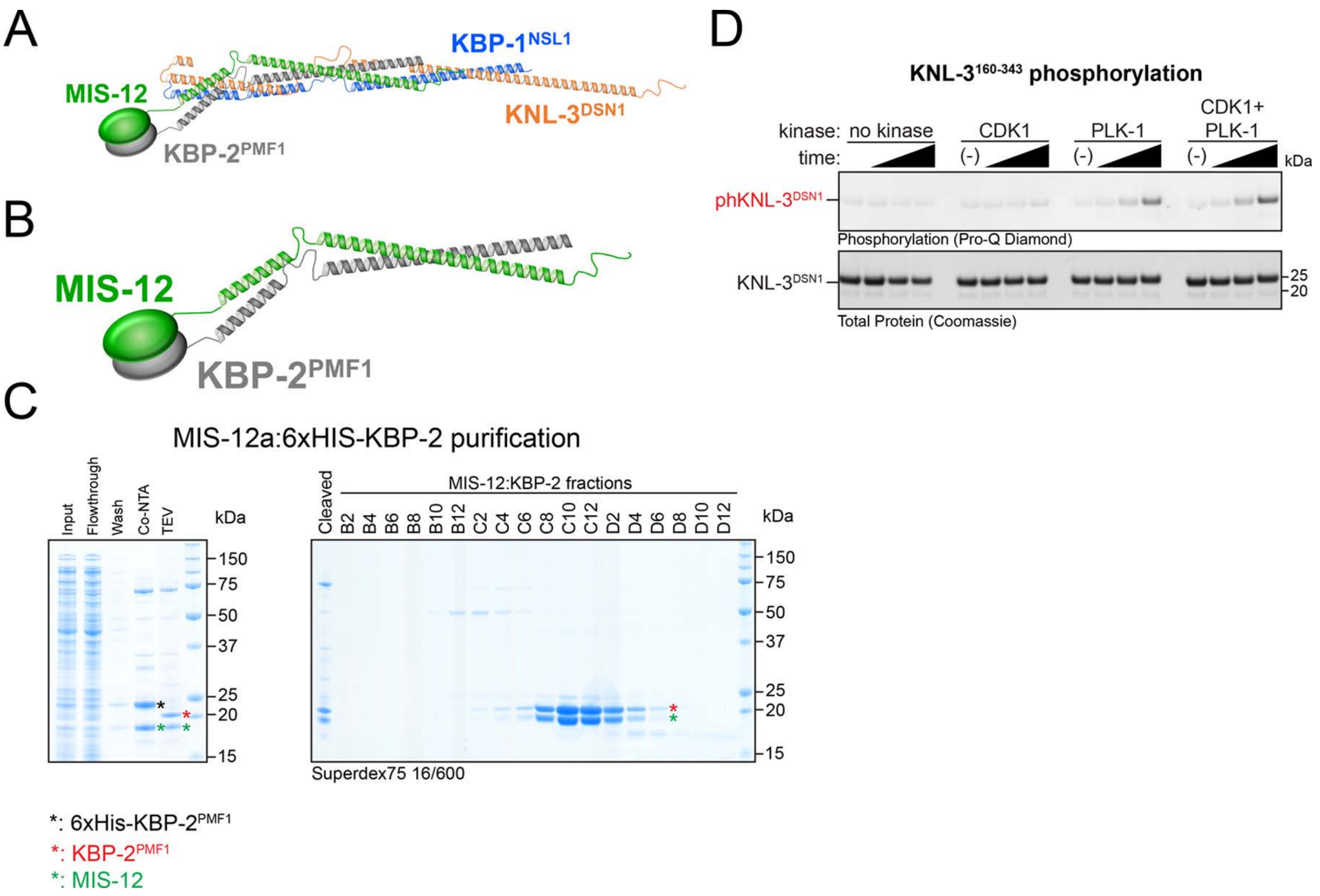


**Fig. S3. Purification of the MIS-12:KBP-2PMF1 dimer.** (A) Schematic of the full MIS-12 complex structure based on an AlphaFold2 prediction. The disordered region of KNL-3$^{DSN1}$ (aa 1-159) is not shown. **(B)** Enlarged image of the MIS-12:KBP-2$^{PMF1}$ complex from (A). **(C)** Biochemical purification of the MIS-12:KBP-2$^{PMF1}$ complex from bacteria. **(D)** Kinase assays were performed with the indicated protein kinases and KNL-3. Time points analysed were 15, 30, and 45 minutes. **(D)** Kinase assays were performed with the indicated protein kinase and KNL-3$^{DSN1}$ as a substrate. Time points analysed were 0, 15, 30, and 45 minutes.

Journal of Cell Science • Supplementary information

**Table S1**. List of phospho-sites identified from in vitro kinase assays

| Substrate Protein | PLK-1 | Cdk1 | Cdk1+PLK-1 |
|---|---|---|---|
| CENP-C | Ser 9 | Ser 9 | Ser 9 |
| | Thr 10 | Thr 10 | Thr 10 |
| | Thr 19 | Thr 19 | Thr 19 |
| | Thr 41 | | Thr 41 |
| | Ser 47 | | Ser 47 |
| | Ser 52 | | Ser 52 |
| | | Ser 66 | Ser 66 |
| | | | Thr 80 |
| | Thr 98 | Thr 98 | Thr 98 |
| | | | Ser 109 |
| | | | Thr 118 |
| | Ser 125 | | Ser 125 |
| | | Ser 127 | Ser 127 |
| | Ser 142 | Ser 142 | Ser 142 |
| | | Ser 146 | Ser 146 |
| | Ser 160 | | Ser 160 |
| | Ser 162 | | Ser 162 |
| | | Thr 163 | Thr 163 |
| | | Ser 166 | Ser 166 |
| | Ser 171 | | Ser 171 |
| | | Ser 174 | Ser 174 |
| | | Ser 177 | Ser 177 |
| | Ser 181 | | Ser 181 |
| | Ser 200 | | Ser 200 |
| | Thr 205 | Thr 205 | Thr 205 |
| | Thr 213 | | Thr 213 |

| Substrate Protein | PLK-1 |
|---|---|
| KBP-2 | Thr 15 |
| | Ser 22 |
| | Thr 64 |
| | Ser 72 |
| | Thr 84 |
| | Ser 114 |
| | Thr 116 |
| | |
| MIS-12 | Ser 90 |
| | Thr 92 |
| | |
| KNL-3 | Ser 177 |
| | Thr 185 |
| | Ser 191 |
| | Ser 225 |
| | Ser 229 |
| | Thr 241 |
| | Ser 258 |
| | Thr 263 |
| | Ser 292 |

Journal of Cell Science • Supplementary information

**Table S2**. *C. elegans* strains used in this study

| | | | |
|---|---|---|---|
| bub-1::linker::gfp; mCherry::histone | FGP202 | *bub-1(syb1134[bub-1::linker::gfp])I; ltIs37 [pAA64; pie-1/mCherry::his-58; unc-119 (+)]IV; unc-119(ed3)III* | This study |
| PLK-1::sfGFP; mCherry::histone | FGP263 | *plk-1(lt18[plk-1::sGFP]::loxp)III; ltIs37 [pAA64; pie-1/mCHERRY::his-58; unc-119 (+)]IV* | doi.org/10.1016/j.devcel.2017.09.019 |
| NDC-80::GFP; mCherry::histone | FGP372 | *ndc-80(lt54[ndc-80::GFP::tev::loxP::3xFlag])IV; ltIs37 [pAA64; pie-1/mCHERRY::his-58; unc-119 (+)]IV* | This study |
| hcp-4(T163A) | FGP669 | *hcp-4(fgp58[hcp-4(T163A)])I* | doi.org/10.7554/eLife.84057 |
| PLK-1::sGFP; mCherry::histone; hcp-4(T163A) | FGP719 | *plk-1(lt18[plk-1::sGFP]::loxp)III; ltIs37 [pAA64; pie-1/mCHERRY::his-58; unc-119 (+)]IV; hcp-4(fgp58[hcp-4(T163A)])I* | doi.org/10.7554/eLife.84057 |
| mScarlet::KNL-3; GFP::histone | FGP722 | *knl-3(dha19 [mScarlet-I^3XFLAG::knl-3] V; ruIs32 [pie-1::GFP::histone + unc-119(+)], unc-119 (ed3)* | This study |
| mScarlet::KNL-3; GFP::histone; hcp-4(T163A) | FGP728 | *knl-3(dha19 [mScarlet-I^3XFLAG::knl-3] V; ruIs32 [pie-1::GFP::histone + unc-119(+)], unc-119 (ed3); hcp-4(fgp58[hcp-4(T163A)])I* | This study |
| NDC-80::GFP; mCherry::histone; hcp-4(T163A) | FGP729 | *ndc-80(lt54[ndc-80::GFP::tev::loxP::3xFlag])IV; ltIs37 [pAA64; pie-1/mCHERRY::his-58; unc-119 (+)]IV; hcp-4(fgp58[hcp-4(T163A)])I* | This study |
| PLK-1::sGFP; mCherry::histone; bub-1(wt) | FGP735 | *plk-1(lt18[plk-1::sGFP]::loxp)III; ; ltIs37 [pAA64; pie-1/mCHERRY::his-58; unc-119 (+)]IV; unc-119(ed3)III;ltSi268[pOD/pTK013; Pbub-1::Bub1 reencoded; cb-unc-119(+)]II* | This study |
| PLK-1::sGFP; mCherry::histone; hcp-4(T163A); bub-1(wt) | FGP736 | *plk-1(lt18[plk-1::sGFP]::loxp)III; ltIs37 [pAA64; pie-1/mCHERRY::his-58; unc-119 (+)]IV; hcp-4(fgp58[hcp-4(T163A)])I; unc-119(ed3)III;ltSi268[pOD/pTK013; Pbub-1::Bub1 reencoded; cb-unc-119(+)]II* | This study |
| PLK-1::sGFP; mCherry::histone; bub-1(T527A) | FGP737 | *plk-1(lt18[plk-1::sGFP]::loxp)III; ltIs37 [pAA64; pie-1/mCHERRY::his-58; unc-119 (+)]IV; unc-119(ed3)III;ltSi1346[pTK070;Pbub-1::BUB-1 T527A reencoded; cb-unc-119(+)]II* | This study |
| PLK-1::sGFP; mCherry::histone; hcp-4(T163A); bub-1(T527A) | FGP738 | *plk-1(lt18[plk-1::sGFP]::loxp)III; ltIs37 [pAA64; pie-1/mCHERRY::his-58; unc-119 (+)]IV; hcp-4(fgp58[hcp-4(T163A)])I; unc-119(ed3)III;ltSi1346[pTK070;Pbub-1::BUB-1 T527A reencoded; cb-unc-119(+)]II* | This study |
| mScarlet::KNL-3; GFP::histone; bub-1(wt) | FGP739 | *knl-3(dha19 [mscarlet-I^3XFLAG::knl-3] V; ruIs32 [pie-1::GFP::histone + unc-119(+)], unc-119 (ed3); unc-119(ed3)III;ltSi268[pOD/pTK013; Pbub-1::Bub1 reencoded; cb-unc-119(+)]II* | This study |
| mScarlet::KNL-3; GFP::histone; hcp-4(T163A); bub-1(wt) | FGP740 | *knl-3(dha19 [mScarlet-I^3XFLAG::knl-3] V; ruIs32 [pie-1::GFP::histone + unc-119(+)], unc-119 (ed3); hcp-4(fgp58[hcp-4(T163A)])I; unc-119(ed3)III;ltSi268[pOD/pTK013; Pbub-1::Bub1 reencoded; cb-unc-119(+)]II* | This study |
| mScarlet::KNL-3; GFP::histone; bub-1(T527A) | FGP741 | *knl-3(dha19 [mScarlet-I^3XFLAG::knl-3] V; ruIs32 [pie-1::GFP::histone + unc-119(+)], unc-119 (ed3); unc-119(ed3)III;ltSi1346[pTK070;Pbub-1::BUB-1 T527A reencoded; cb-unc-119(+)]II* | This study |
| mScarlet::KNL-3; GFP::histone; hcp-4(T163A); bub-1(T527A) | FGP742 | *knl-3(dha19 [mscarlet-I^3XFLAG::knl-3] V; ruIs32 [pie-1::GFP::histone + unc-119(+)], unc-119 (ed3); hcp-4(fgp58[hcp-4(T163A)])I; unc-119(ed3)III;ltSi1346[pTK070;Pbub-1::BUB-1 T527A reencoded; cb-unc-119(+)]II* | This study |

| | | | |
|---|---|---|---|
| NDC-80::GFP; mCherry::histone; bub-1(wt) | FGP803 | *ndc-80(lt54[ndc-80::GFP::tev::loxP::3xFlag])IV; ltIs37 [pAA64; pie-1/mCHERRY::his-58; unc-119 (+)]IV; Pbub-1::Bub1 reencoded; cb-unc-119(+)]II* | This study |
| NDC-80::GFP; mCherry::histone; hcp-4(T163A); bub-1(wt) | FGP804 | *ndc-80(lt54[ndc-80::GFP::tev::loxP::3xFlag])IV; ltIs37 [pAA64; pie-1/mCHERRY::his-58; unc-119 (+)]IV; hcp-4(fgp58[hcp-4(T163A)])I; unc-119(ed3)III;ltSi268[pOD/pTK013; Pbub-1::Bub1 reencoded; cb-unc-119(+)]II* | This study |
| NDC-80::GFP; mCherry::histone; bub-1(T527A) | FGP805 | *ndc-80(lt54[ndc-80::GFP::tev::loxP::3xFlag])IV; ltIs37 [pAA64; pie-1/mCHERRY::his-58; unc-119 (+)]IV;ltSi1346[pTK070;Pbub-1::BUB-1 T527A reencoded; cb-unc-119(+)]II* | This study |
| NDC-80::GFP; mCherry::histone; hcp-4(T163A); bub-1(T527A) | FGP806 | *ndc-80(lt54[ndc-80::GFP::tev::loxP::3xFlag])IV; ltIs37 [pAA64; pie-1/mCHERRY::his-58; unc-119 (+)]IV; hcp-4(fgp58[hcp-4(T163A)])I; unc-119(ed3)III; ltSi1346[pTK070;Pbub-1::BUB-1 T527A reencoded; cb-unc-119(+)]II* | This study |
| bub-1::linker::gfp; mCherry::histone; hcp4(T163A) | FGP815 | *bub-1(syb1134[bub-1::linker::gfp])I; ltIs37 [pAA64; pie-1/mCherry::his-58; unc-119 (+)]IV; unc-119(ed3)III; hcp-4(fgp58[hcp-4(T163A)])I* | This study |
| GFP::CDC-20; mCherry::histone | OD2591 | *ltSi814[pPLG047; Pfzy-1::gfp::fzy-1::fzy-1 3'UTR; cb-unc-119(+)]I; unc-119(ed3)III?;; ltIs37 [pAA64; pie-1/mCHERRY::his-58; unc-119 (+)] IV* | doi.org/10.1101/gad.302067.117 |
| GFP::CDC-20; mCherry::histone; hcp-4(T163A) | FGP829 | *ltSi814[pPLG047; Pfzy-1::gfp::fzy-1::fzy-1 3'UTR; cb-unc-119(+)]I; unc-119(ed3)III?;; ltIs37 [pAA64; pie-1/mCHERRY::his-58; unc-119 (+)] IV; hcp-4(fgp58[hcp-4(T163A)])I* | This study |

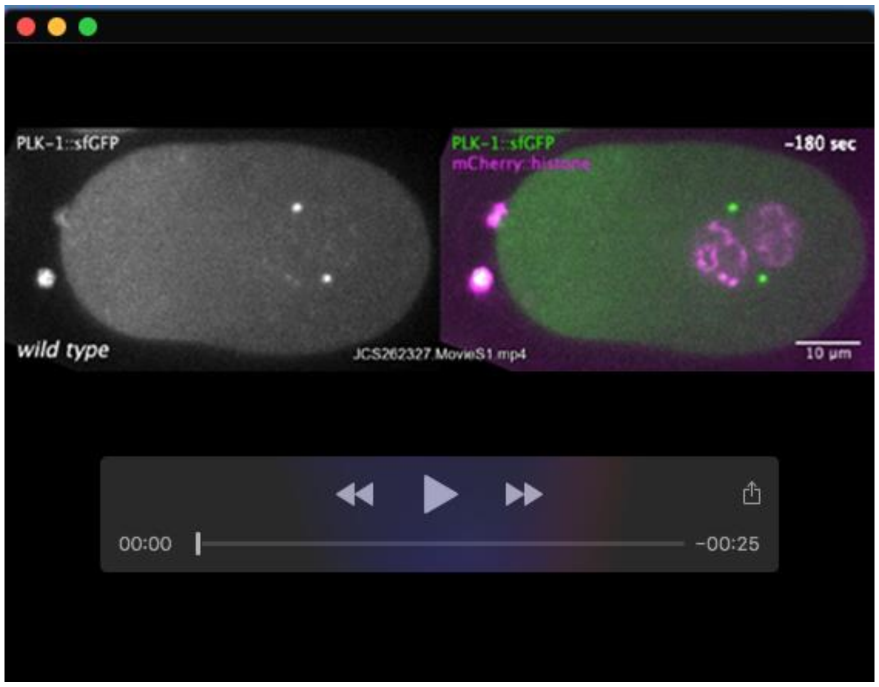


**Movie 1. Embryo expressing PLK-1::sfGFP and mCherry::histone was imaged throughout mitosis.** Movie corresponds to 'wild type' panels in Fig. 1B.

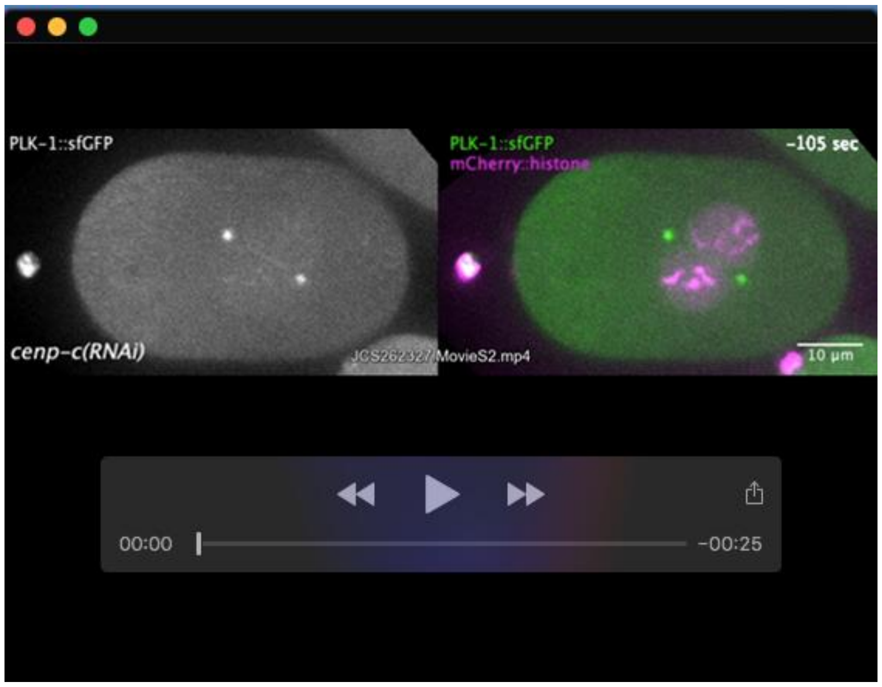


**Movie 2. Embryo expressing PLK-1::sfGFP and mCherry::histone was imaged throughout mitosis.** Movie corresponds to '*cenp-c(RNAi)*' panels in Fig. 1B.

Journal of Cell Science • Supplementary information

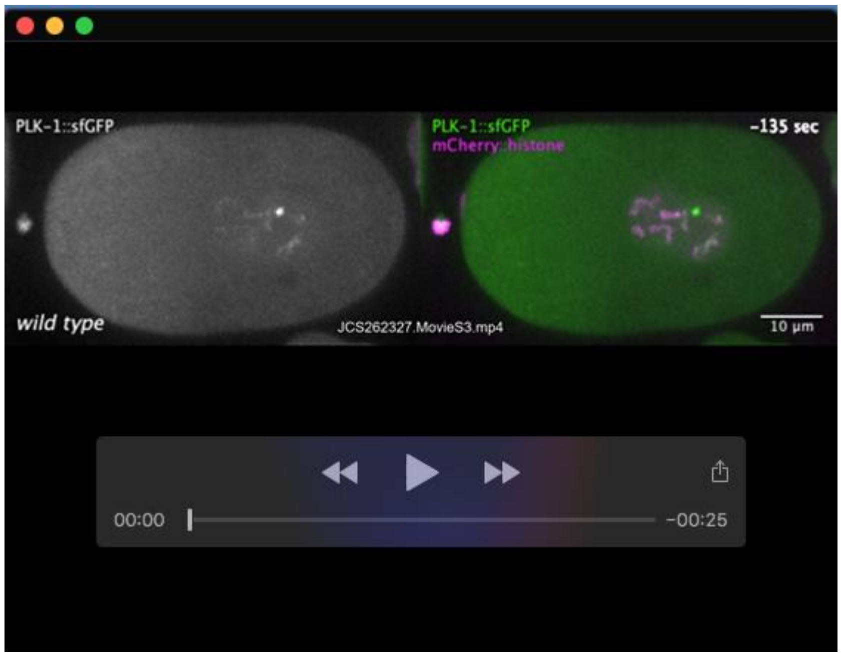


**Movie 3. Embryo expressing PLK-1::sfGFP and mCherry::histone was imaged throughout mitosis.** Movie corresponds to 'wild type' panels in Fig. 1F.

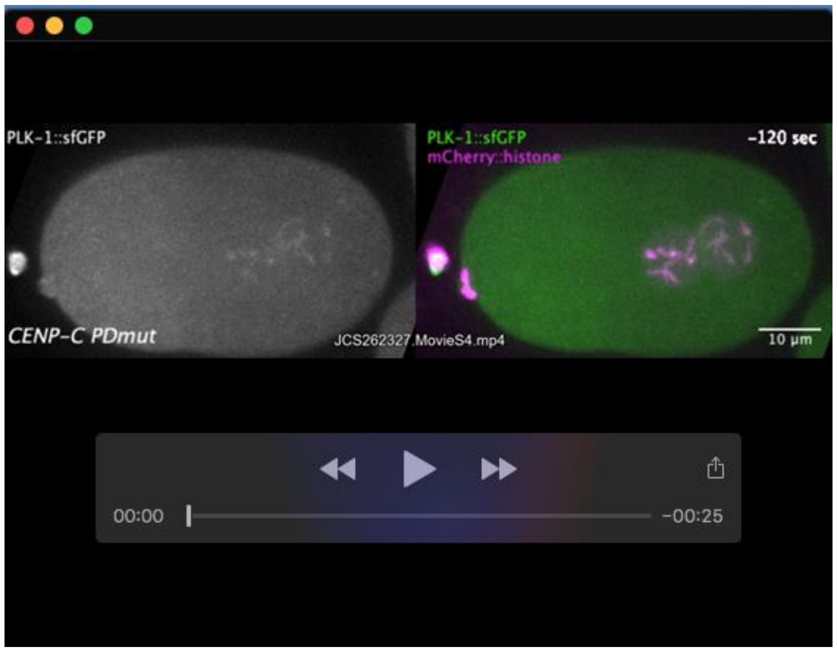


**Movie 4. Embryo expressing PLK-1::sfGFP and mCherry::histone was imaged throughout mitosis.** Movie corresponds to '*CENP-C PD$^{mut}$*' panels in Fig. 1F.

Journal of Cell Science • Supplementary information

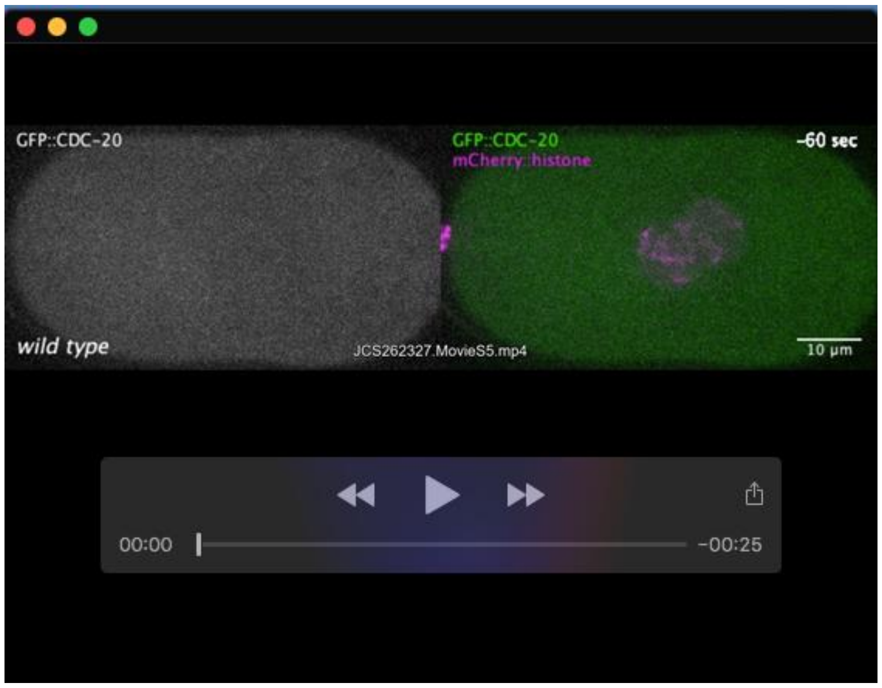


**Movie 5. Embryo expressing GFP::CDC-20 and mCherry::histone was imaged throughout mitosis.** Movie corresponds to '*wild type*' panels in Fig. 2A.

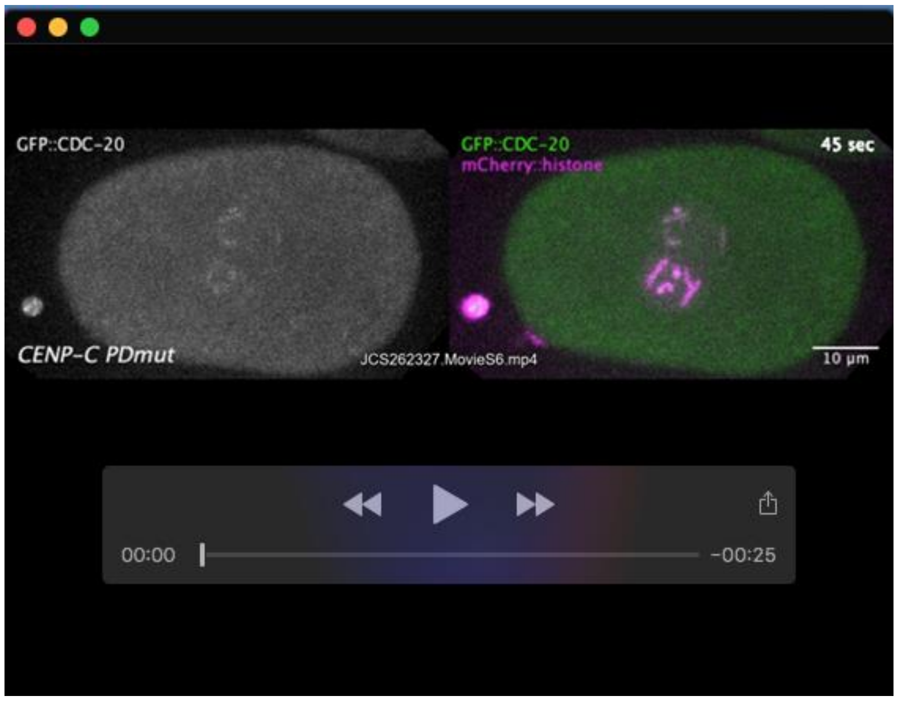


**Movie 6. Embryo expressing GFP::CDC-20 and mCherry::histone was imaged throughout mitosis.** Movie corresponds to '*CENP-C PD$^{mut}$*' panels in Fig. 2A.

Journal of Cell Science • Supplementary information

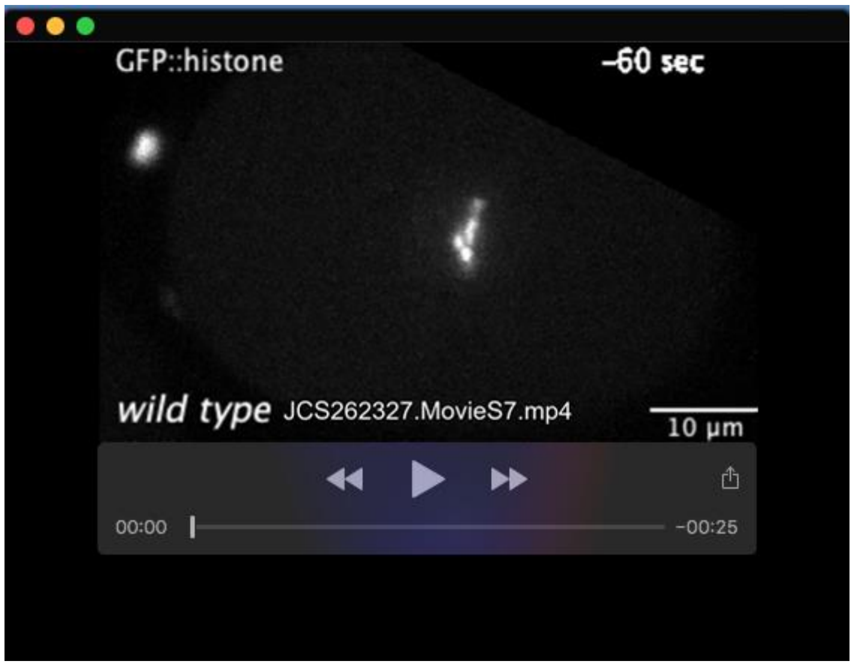


**Movie 7. Embryo expressing GFP::histone was imaged throughout mitosis.** Movie corresponds to '*wild type*' panels in Fig. 2D. The strain used here was FGP722.

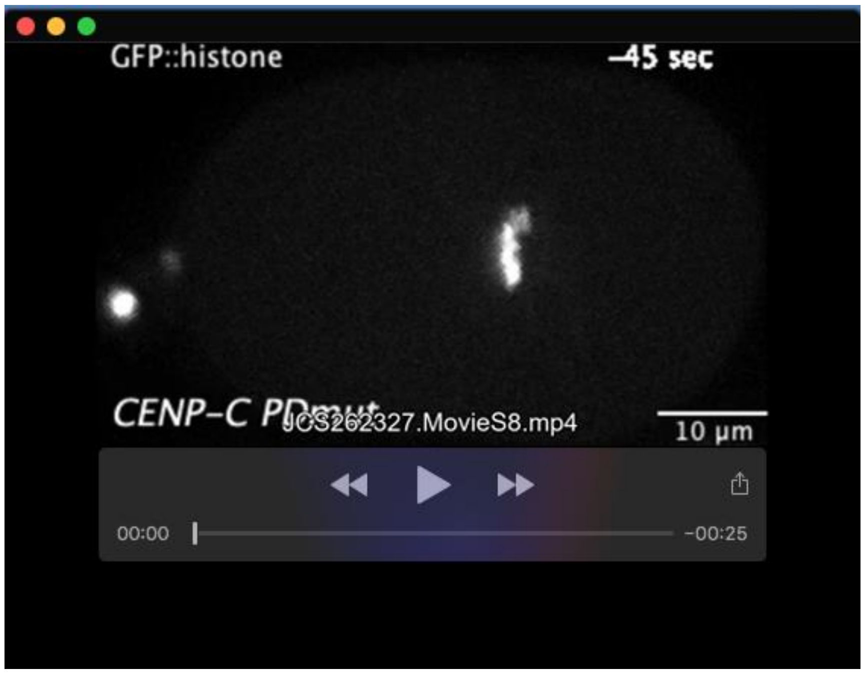


**Movie 8. Embryo expressing GFP::histone was imaged throughout mitosis.** Movie corresponds to '*CENP-C PD*$^{mut}$' panels in Fig. 2D. The strain used here was FGP728.

Journal of Cell Science • Supplementary information

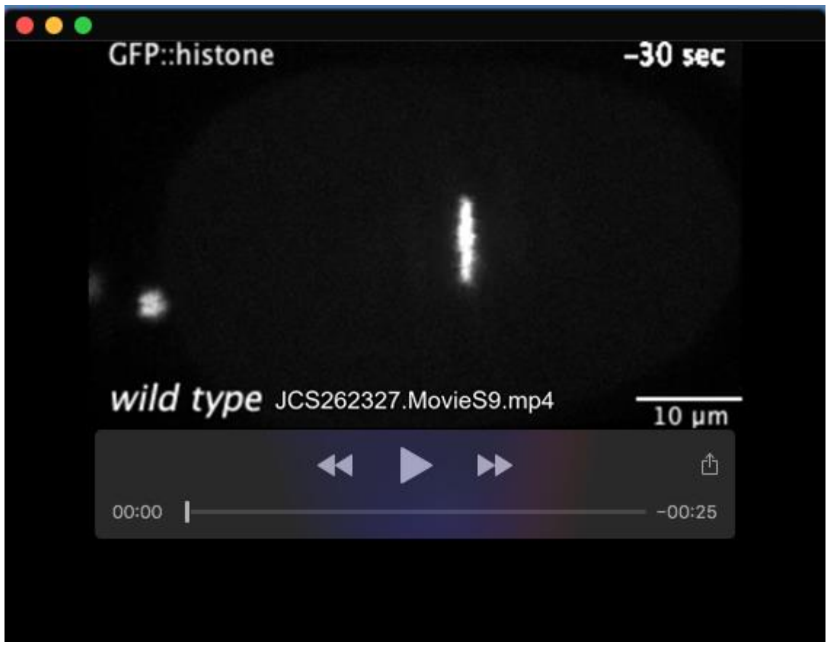


**Movie 9. Embryo expressing GFP::histone was imaged throughout mitosis.** Movie corresponds to '*wild type*' panels in Fig. 2E. The strain used here was FGP722.

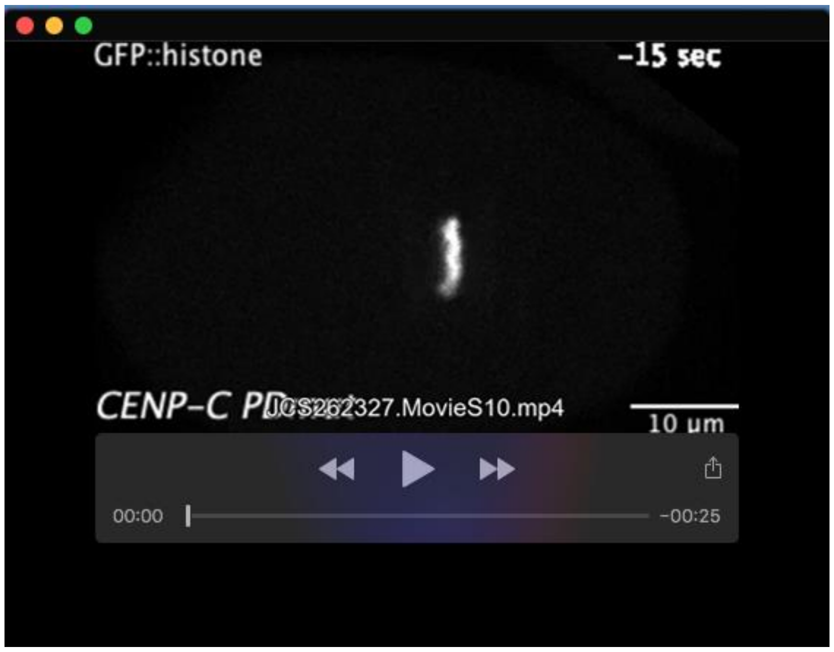


**Movie 10. Embryo expressing GFP::histone was imaged throughout mitosis.** Movie corresponds to '*CENP-C PD$^{mut}$*' panels in Fig. 2E. The strain used here was FGP728.

Journal of Cell Science • Supplementary information

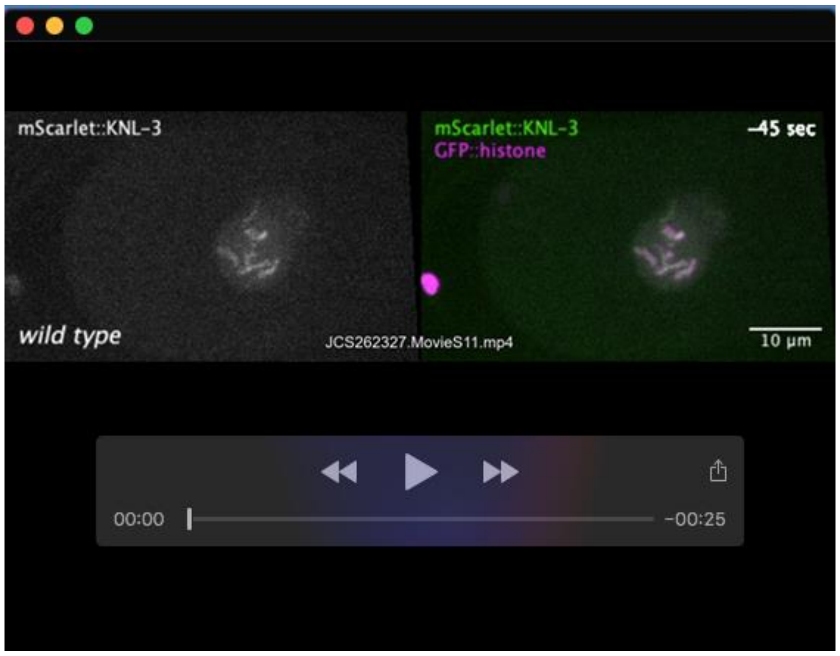


**Movie 11. Embryo expressing Scarlet::KNL-3 and GFP::histone was imaged throughout mitosis.** Movie corresponds to '*wild type*' panels in Fig. 3B.

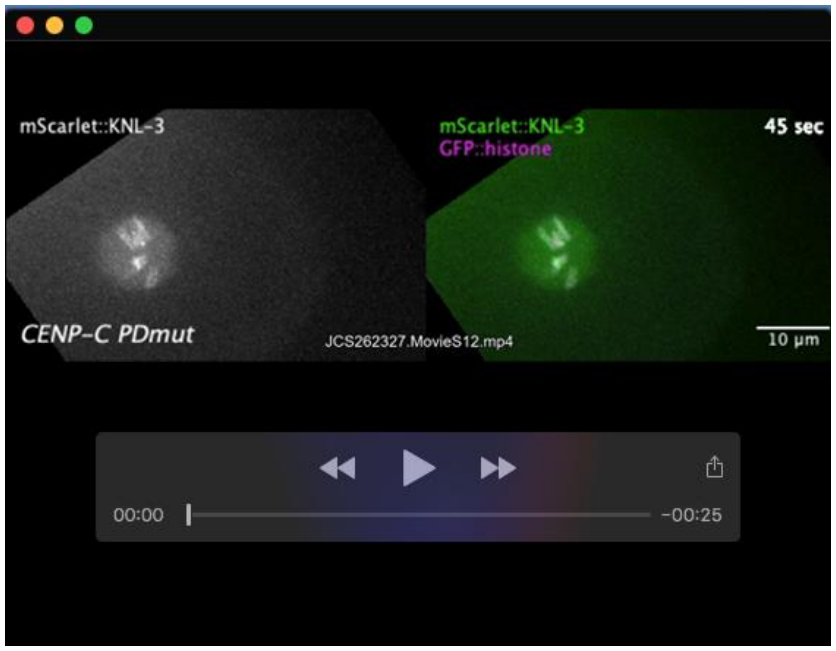


**Movie 12. Embryo expressing Scarlet::KNL-3 and GFP::histone was imaged throughout mitosis.** Movie corresponds to '*CENP-C PD*$^{mut}$' panels in Fig. 3B.

Journal of Cell Science • Supplementary information

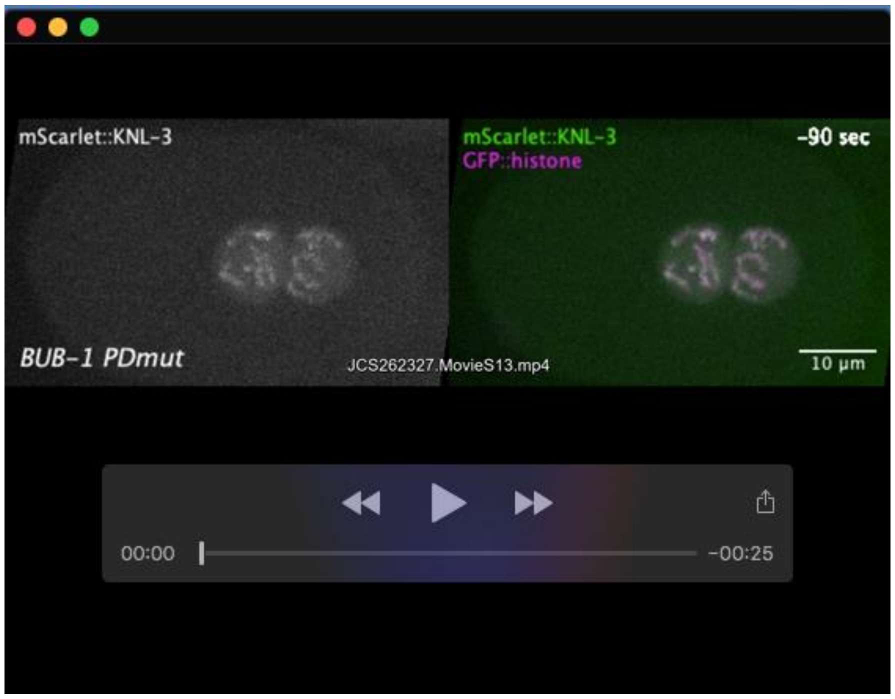


**Movie 13. Embryo expressing Scarlet::KNL-3 and GFP::histone was imaged throughout mitosis.** Movie corresponds to ‘*BUB-1 PD$^{mut}$*’ panels in Fig. 3B.

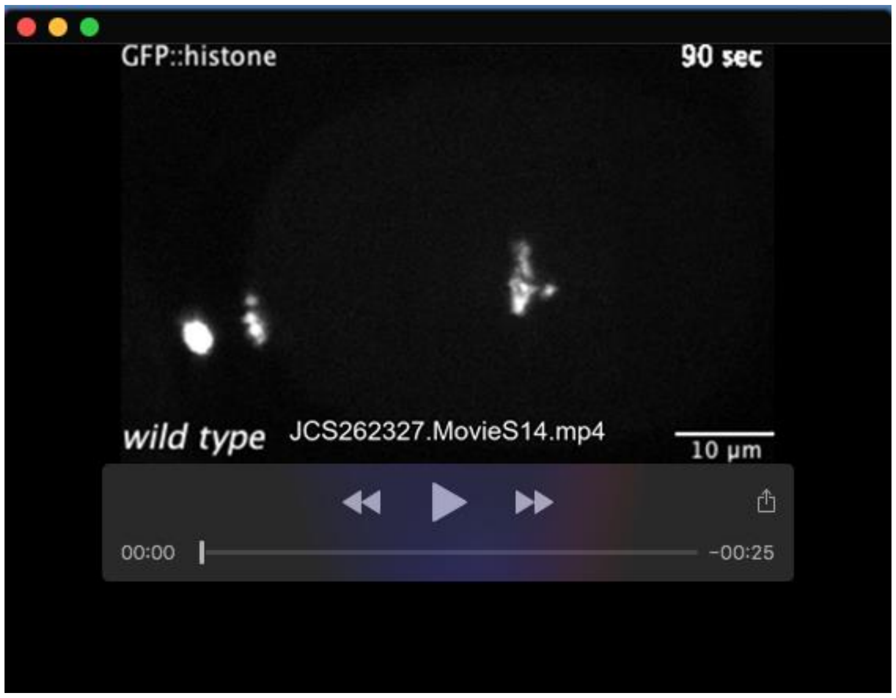


**Movie 14. Embryo expressing GFP::histone was imaged throughout mitosis.** Movie corresponds to ‘*wild type*’ panels in Fig. 4A.

Journal of Cell Science • Supplementary information

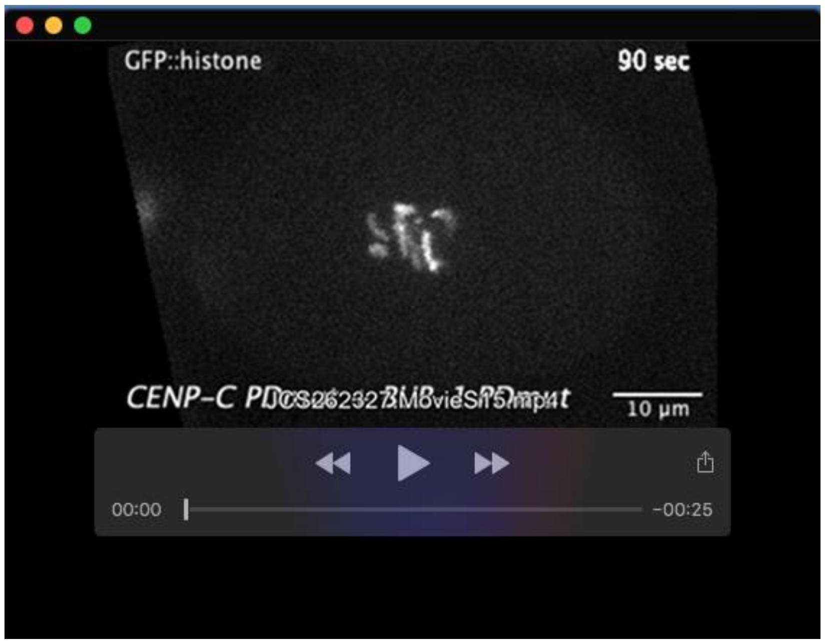


**Movie 15. Embryo expressing GFP::histone was imaged throughout mitosis.** Movie corresponds to '*CENP-C PD*$^{mut}$ + *BUB-1 PD*$^{mut}$' panels in Fig. 4A.

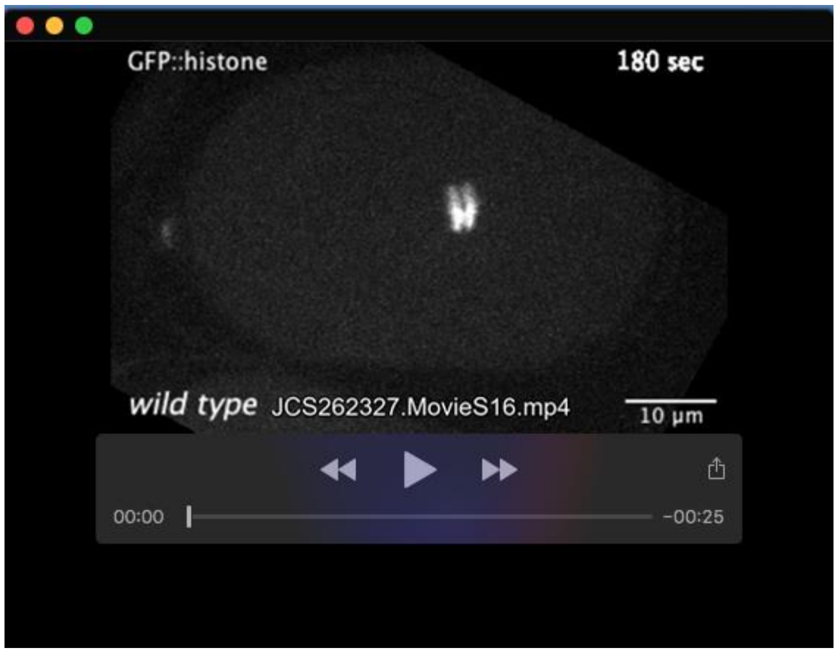


**Movie 16. Embryo expressing Scarlet::KNL-3 and GFP::histone was imaged throughout mitosis.** Movie corresponds to '*wild type*' panels in Fig. 4B.

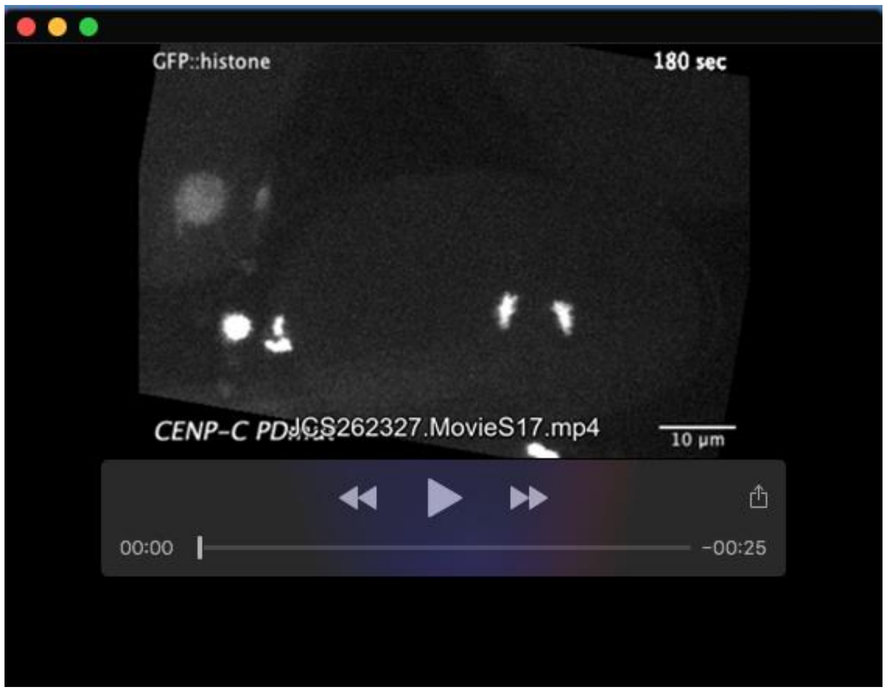


**Movie 17. Embryo expressing Scarlet::KNL-3 and GFP::histone was imaged throughout mitosis.** Movie corresponds to '*CENP-C PD$^{mut}$*' panels in Fig. 4B.

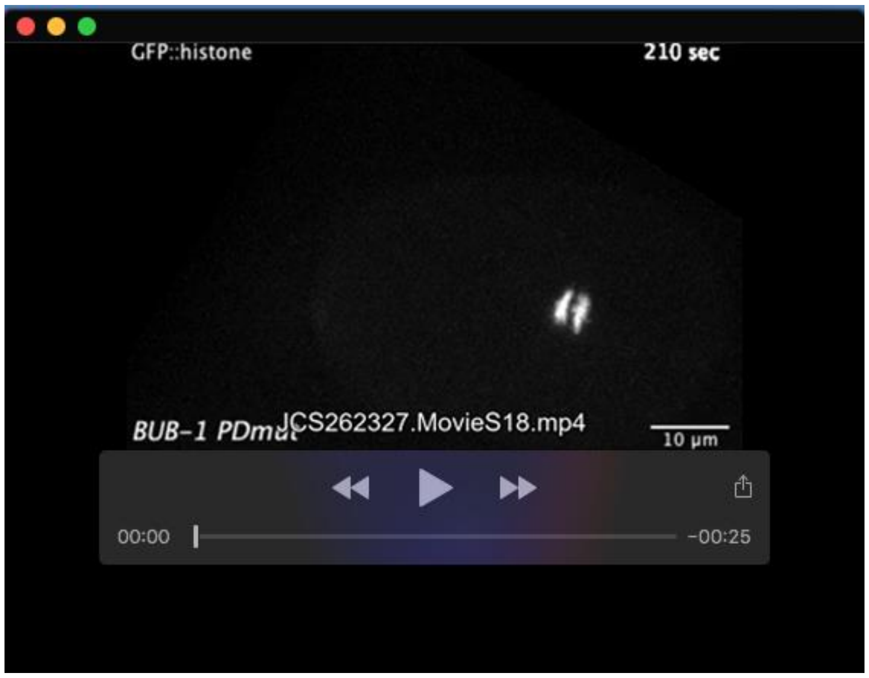


**Movie 18. Embryo expressing GFP::histone was imaged throughout mitosis.** Movie corresponds to '*BUB-1 PD$^{mut}$*' panels in Fig. 4B.

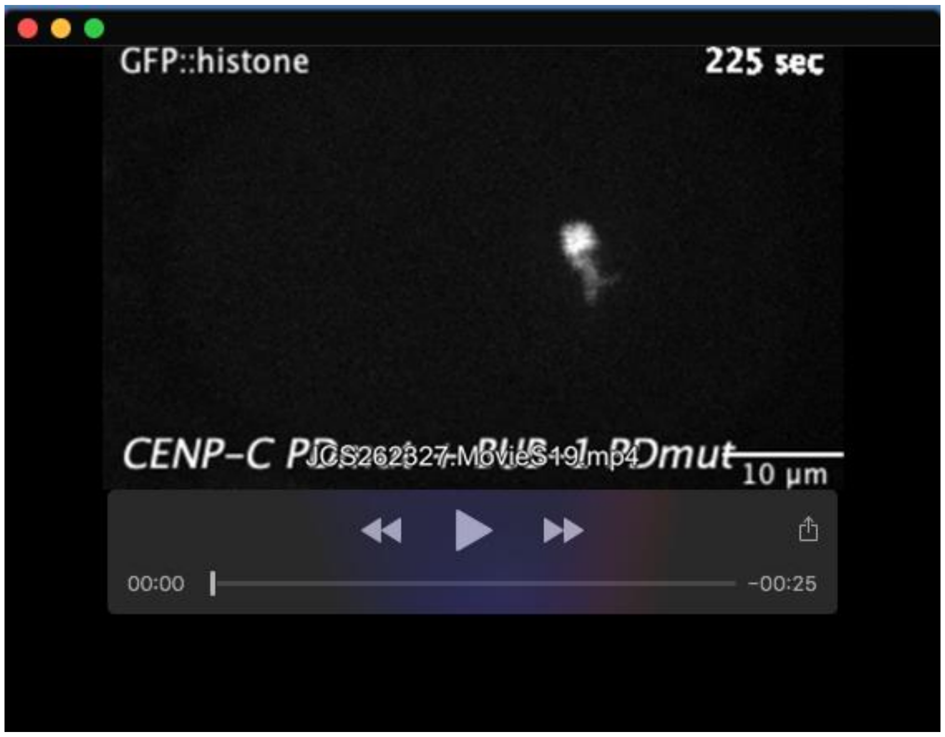


**Movie 19. Embryo expressing GFP::histone was imaged throughout mitosis.**
Movie corresponds to '*CENP-C PD*$^{mut}$ + *BUB-1 PD*$^{mut}$' panels in Fig. 4B.

Journal of Cell Science • Supplementary information
