# Supplementary material for: CENP-C-targeted PLK-1 regulates kinetochore function in C. elegans embryos
Source: J Cell Sci. 2024 Nov 28;137(22):jcs262327. doi: 10.1242/jcs.262327 (PMC11634037; doi:10.1242/jcs.262327)
Supplement: Supplementary file 1 [file joces-137-262327_review_history.pdf]

CENP-C-targeted PLK-1 regulates kinetochore function in *C. elegans* embryos

Laura Bel Borja, Samuel JP Taylor, Flavie Soubigou and Federico Pelisch
DOI: 10.1242/jcs.262327

Editor: Renata Basto

Review timeline
Original submission: 6 June 2024
Editorial decision: 12 July 2024
First revision received: 9 August 2024
Accepted: 20 September 2024

Original submission

First decision letter

MS ID#: JOCES/2024/262327

MS TITLE: CENP-C-targeted PLK-1 regulates kinetochore function in *C. elegans* embryos

AUTHORS: Laura Bel Borja, Samuel JP Taylor, Flavie Soubigou, and Federico Pelisch

We have now reached a decision on the above manuscript.

To see the reviewers' reports and a copy of this decision letter, please go to: https://submit-jcs.biologists.org and click on the 'Manuscripts with Decisions' queue in the Author Area. (Corresponding author only has access to reviews.)

As you will see, the reviewers raise a number of substantial criticisms that prevent me from accepting the paper at this stage. They suggest, however, that a revised version might prove acceptable, if you can address their concerns. If you think that you can deal satisfactorily with the criticisms on revision, I would be pleased to see a revised manuscript. We would then return it to the reviewers.

Please ensure that you clearly highlight all changes made in the revised manuscript. Please avoid using 'Tracked changes' in Word files as these are lost in PDF conversion.

I should be grateful if you would also provide a point-by-point response detailing how you have dealt with the points raised by the reviewers in the 'Response to Reviewers' box. Please attend to all of the reviewers' comments. If you do not agree with any of their criticisms or suggestions please explain clearly why this is so.

Reviewer 1

Advance summary and potential significance to field

The essential mitotic kinase PLK-1 is recruited to multiple spatial locations during cell division, with different pools of PLK-1 executing different functions. Using the early C. elegans embryo as a model, Bel Borja et al. study the function of inner-kinetochore localized PLK-1, whose function during mitosis has not been previously characterized. The authors find that PLK-1 bound to CENP-C

© 2024. Published by The Company of Biologists under the terms of the Creative Commons Attribution License (https://creativecommons.org/licenses/by/4.0/).

plays an important role in chromosome segregation and is involved in controlling recruitment of MIS12 and NDC80 to the outer kinetochore. They also show that dual inhibition of the CENP-C and BUB-1 bound pools of PLK-1 has a synergistic negative impact on chromosome segregation. The data overall are nice; however, several points need to be addressed before publication. If possible, making some connection between CENP-C-bound PLK-1 and control of outer kinetochore assembly would strengthen the work.

Comments for the author

The essential mitotic kinase PLK-1 is recruited to multiple spatial locations during cell division, with different pools of PLK-1 executing different functions. Using the early C. elegans embryo as a model, Bel Borja et al. study the function of inner-kinetochore localized PLK-1, whose function during mitosis has not been previously characterized. The authors find that PLK-1 bound to CENP-C plays an important role in chromosome segregation and is involved in controlling recruitment of MIS12 and NDC80 to the outer kinetochore. They also show that dual inhibition of the CENP-C and BUB-1 bound pools of PLK-1 has a synergistic negative impact on chromosome segregation. The data overall are nice; however, several points need to be addressed before publication. If possible, making some connection between CENP-C-bound PLK-1 and control of outer kinetochore assembly would strengthen the work.

Major Points/Queries:

- Inhibiting PLK-1 binding to CENP-C leads to slightly faster mitotic timing and spindle snapping during anaphase; it also leads to increased recruitment of of NDC80 and MIS-12 complexes to kinetochores. Is there any connection between these phenotypes? Moreover, does CENP-C PDmut affect CENP-C localization itself, or localization of KNL-1? Mapping which kinetochore components get hyper-recruited with CENP-C PDmut (testing components from centromeric chromatin to the microtubule interface) would help delineate at which step CENP-C bound PLK-1 acts in regulating kinetochore assembly.
- Fig 3: Phosphorylation of KBP-2 by PLK-1 is clearly shown- were any phospho-sites identified that regulate its interaction with CENP-C? Or was there any mass spec analysis conducted of the phosphorylated proteins? While a detailed mechanism is not necessary for publication, any connection between potential PLK-1-mediated phosphorylation (e.g. of CENP-C itself or of the MIS-12 complex) and the observed limiting of outer kinetochore protein recruitment would greatly elevate the impact of the work.

Minor Points:

- How efficient/effective is the feeding RNAi for CENP-C or BUB-1? A control showing knockdown of the endogenous protein (either imaging of an endogenously tagged strain in the presence of RNAi or a western blot showing depletion of the endogenous protein) is needed to confirm that the RNAi worked.
- Line 156: Typo, “to focus or further analysis” -> “to focus our further analysis”
- Line 163: Possible incomplete sentence
- Line 211: Typo, “yeloow” to “yellow”
- Line 230: Figure 2 legend for 2E is incorrect (chromosome distance, not centrosome to centrosome distance assay) and legend for 2F is missing (centrosome separation assay).
- Figure 1B: Images are grainy/pixelated, looks like an import issue. Please upload some clearer images.
- Figure 2: n for 2D-F are missing, please show. Also the fluorophores for that experiment aren’t labeled.
- Figure 3B: n is missing, please show.
- Fig 4D- would be nice to have images of PLK-1 alongside the quantification.
- S1A: Decrease in PLK-1 with IF in the CENP-C PDmut looks much stronger than the in vivo imaging- are the IF images quantifiable? Reason for difference between IF and in vivo imaging?
- S1B- would be nice to have BUB-1 quantification (the images don’t look appreciably different but would be nice to have quantification to show that they’re the same).
- S2B would be nice to have quantification for NDC80 recruitment (just at metaphase would be fine just to go with the images showing increase with the CENP-C mutant)
- Strain table: There appear to be strains missing- I don’t see the strain with both HCP-4 PDmut

© 2024. Published by The Company of Biologists under the terms of the Creative Commons Attribution License (https://creativecommons.org/licenses/by/4.0/).

<u>Reviewer 2</u>

Advance summary and potential significance to field

The Polo-like kinase 1 (Plk1) is instrumental for mitotic entry and progression. In particular, Plk1 is recruited to the kinetochores to regulate chromosome segregation during meiosis and mitosis. Understanding how Plk1 gets recruited to the kinetochores and identifying its critical targets at this location is essential for deciphering Plk1's function in chromosome segregation and maintaining genome integrity.

Previous work revealed that Plk1 targeting at the kinetochore depends on BUB1 in the outer kinetochore and the constitutive centromere-associated network (CCAN) in the inner kinetochore. Plk1 recruited by BUB1 regulates CDC20 phosphorylation, the spindle assembly checkpoint, and mitosis timing. However the role of PLK1 recruited to the inner kinetochore needs to be clarified.

Here, the authors use the C. elegans embryo to dissect the mechanisms and function of PLK1 recruitment to the inner kinetochore. In C. elegans, HCP-4/CENP-C is the unique CCAN component. In previous work the authors showed that PLK1 is recruited to the kinetochores via BUB-1 and HCP-4/CENP-C phosphorylated at unique Polo-docking sites during meiosis. They showed that abrogating PLK-1 recruitment to the kinetochores by combining point mutations on the Polo-docking sites of HCP-4/CENP-C and BUB-1 causes severe chromosome segregation defects.
Here, they extended their observations to mitosis. They show that HCP-4/CENP-C, phosphorylated at a unique Polo-docking site, recruits PLK-1 to the inner kinetochore during mitosis. Mutation of the Polo-docking site of HCP-4/CENP-C diminishes PLK-1 levels on kinetochores after NEBD. This pool of PLK-1 is not involved in CDC20 function, spindle assembly checkpoint, and mitotic timing regulation. Instead preventing PLK-1 recruitment to HCP-4/CENP-C causes excessive MIS12 complex recruitment, defects in chromosome congression to the metaphase plate, and an imbalance in microtubule pulling forces during anaphase. As in meiosis, combining Polo-docking site mutations of BUB-1 and HCP-4 causes severe chromosome segregation defects during mitosis.
Overall, this study provides additional information about PLK1 function at the kinetochore. Most experiments are well-executed and relatively convincing, with some exceptions (see below). However, the phenotypic analysis of the HCP-4/CENP-C mutant could be further extended, or at least some observations should be consolidated.

Comments for the author

Major points:

The authors show in Figure 2E that chromosomes separate prematurely in the CENP-C PD mutant; however, this phenotype is not apparent in Figure 1D. How do the authors explain this difference?

Combining mutations of the Polo-docking sites of BUB-1 and HCP-4/CENP-C abrogates PLK1 recruitment to the kinetochores during meiosis, causing major chromosome segregation defects. Therefore, how do the authors exclude the possibility that the defects they see during mitosis are not a consequence of the meiotic defects? They should at least discuss that point.

The authors should better characterize the phenotype of the CENP-C PD mutant by monitoring other kinetochore components (MIS12 subunits and others). How specific is the phenotype? Is only MIS12 deregulated? As for the phenotypic analysis, not all embryos have the same length, so the authors should normalize the centrosome-to-centrosome and chromosome distance to embryo length (Figures 2D and 2F).

Most of Figure 2B, where the authors show that the HCP-4 mutant does not impact CDC-20, could be moved to the supplementary material.

The authors show that PLK-1 phosphorylates the MIS12 subunit in vitro, but this potentially interesting observation has not been followed up, leaving the reader with little more information.

© 2024. Published by The Company of Biologists under the terms of the Creative Commons Attribution License (https://creativecommons.org/licenses/by/4.0/).

Minor points:
- Some sentences still need to be finished, and some words must be included! lane 86, “To answer this question, we monitored?”
- The drawing in Figure 1A needs to be more accurate. During anaphase, the mitotic spindle is asymmetrically positioned, resulting in a larger anterior and a smaller posterior cell. Embryos in positions 5, 6, and 7 should be turned to 180°C.
- The drawing in Figure S2A would be helpful in the main Figures for showing the HCP-4/CENP-C position at the kinetochore.
- The authors should be consistent with the nomenclature. Sometimes, they use the C. elegans nomenclature and, at other places, the human one. It is confusing. They could present the human and C.
elegans nomenclature in a short table at the beginning or the double nomenclature throughout the manuscript, as done for KNL-3DSN1. Generally speaking, it is preferable to use the C. elegans nomenclature to talk about the proteins of this organism.
- Lane 120: add we imaged the endogenously tagged “MIS12 subunit” KNL-3/DSN1 subunit.
- Figure 2F needs to be described in the figure legend.
- Add timings in Panels 1B, 1D, 2A, 2D, 2E, 3A, S1B
- The number of experiments conducted (N) and embryos analyzed (n) is not always clearly indicated.
- The overall quality of some figures could be improved (e.g., Fig 1B, 1D).
- Scale bars are missing Figure 4C

<u>Reviewer 3</u>

Advance summary and potential significance to field

Previously, it was shown in mammals that Polo-like kinase 1 is targeted to the inner kinetochore and the outer kinetochore by different mechanisms. However, whether these two subpopulations of kinetochore PLK1 play distinct roles is not known. In this manuscript, Borja et.al. address this question using C. elegans as a model system. This work builds nicely on a previous study by the same group (Taylor et.al., eLife) establishing requirements for PLK-1 targeting to oocyte chromosomes. In this manuscript, they leverage mutants characterized in the previous study to disrupt the targeting of inner kinetochore PLK-1 in mitosis, allowing them to reveal important functions for this population of PLK-1, separable from outer-kinetochore-targeted PLK-1. Overall, I found these findings to be interesting, generally well-supported, and of value to the field.

Comments for the author

Previously, it was shown in mammals that Polo-like kinase 1 is targeted to the inner kinetochore and the outer kinetochore by di:erent mechanisms. However, whether these two subpopulations of kinetochore PLK1 play distinct roles is not known. In this manuscript, Borja et.al. address this question using *C. elegans* as a model system. This work builds nicely on a previous study by the same group (Taylor et.al., *eLife*) establishing requirements for PLK-1 targeting to oocyte chromosomes. In this manuscript, they leverage mutants characterized in the previous study to disrupt the targeting of inner kinetochore PLK-1 in mitosis, allowing them to reveal important functions for this population of PLK-1, separable from outer-kinetochore-targeted PLK-1. Overall, I found these findings to be interesting, well-supported, and of value to the field.

I have a few suggestions that I think would improve the manuscript prior to publication; although most of these are text changes aimed at improving clarity, in a few cases additional quantification should be added to improve the rigor of the manuscript.

Specific points:
- Abstract: Most proteins are referred to with the mammalian nomenclature in the abstract, with no dash (i.e. PLK1 instead of PLK-1). However, there are instances where PLK-1 and CDC-20 are written with dashes. Make the nomenclature consistent (or be sure to only use the dash when you are referring to the worm homolog).

© 2024. Published by The Company of Biologists under the terms of the Creative Commons Attribution License (https://creativecommons.org/licenses/by/4.0/).

- Line 55: it is stated that there is a reduction in PLK-1 levels after CENP-C depletion, but from the figure it looks like this reduction is only in the chromosomal levels (PLK- 1 is appears to be at similar levels at the centrosomes). Modify the sentence to change the phrase to "reduction in chromosomal PLK-1 levels" for clarity.
- Line 60-61: In this manuscript the CENP-C point mutant is described as "CENP-C PD^{mut}" , whereas in the previous paper where this mutant was first described, it was referred to as "*hcp-4* ^{T163A}". It is fine to use a di:erent notation in this manuscript but I think it would be easier for the reader to compare and synthesize the findings from the two manuscripts if, the first time this mutant is mentioned, you define it the way you did previously (e.g. "…a CENP-C point mutant that disrupts its polo docking motif (*hcp-4* ^{T163A}, hereafter referred to as CENP-C PD^{mut})"…)
- The schematic in Figure 2E does not show any interpolar microtubules in the spindle midzone. I suggest using the same spindle diagram as in Figure 1A, for consistency.
- Although this would already be clear to kinetochore a:icionados, I would edit the sentence on line 120 to make it clear that KNL-3^{DSN1} is a component of the Mis12 complex (novice readers may not know this and may be confused as to why you are looking at this protein).
- Although most of the conclusions in the manuscript are well quantified, Figure 3D, S1A, S1B, S2B, and S2C show only a single image for each condition. The authors should provide some sort of quantification to better support these findings. At a minimum, the authors should state in the figure legend how many oocytes were analyzed and looked like the representative image (e.g. "x/x oocytes imaged had increased KNL-3/NDC-80 intensity in the mutant…").

Typos:

- Line 10: should be "the nuclear envelope"
- Line 67: "Figure D" should be "Figure 1D"
- Line 129: delete "in"
- Line 211: yellow is misspelled
- Line 86: I think you are missing a word at the end of this line - add "assessed"?
- Line 96: "lead" should be "led"
- Line 101: a brown arrow is mentioned but it looks red to me on the figure. May just be an issue with my screen, but consider changing the color of the arrow on the figure.
- Line 156: "or" should be "our"
- Line 161: refers to "Figure 4X" - this should be "4D"
- Line 162: I think the word "that" should be deleted?
- Line 182: "in" should be "on"

First revision

Author response to reviewers' comments

REVIEWER 1

Major Points/Queries:

Inhibiting PLK-1 binding to CENP-C leads to slightly faster mitotic timing and spindle snapping during anaphase; it also leads to increased recruitment of NDC80 and MIS-12 complexes to kinetochores. Is there any connection between these phenotypes?

Unfortunately, we do not have the answer to this question at this stage. We could propose some correlations and hypotheses, but we intend to fully address the mechanisms underlying these phenotypes in future work. Once we get the full picture of all kinetochore components being mis-

© 2024. Published by The Company of Biologists under the terms of the Creative Commons Attribution License (https://creativecommons.org/licenses/by/4.0/).

regulated in the CENP-C PD^{mut}, we will be able to formulate a more informed hypothesis and test it in vivo and in vitro. The structure of *C. elegans* kinetochore is not known and will likely differ from the mammalian structures. In fact, most of the interaction nodes within the KMN network will be different as *C. elegans* lacks the regions identified within several components of the MIS12 complex, NDC80 complex, and KNL1. Therefore, we cannot rely on mammalian structures for this and an important first step will be to get structural data for kinetochore components.

At this stage, we believe we present sufficient evidence to establish that kinetochore function is deregulated in the CENP-C PD mutant and that the phenotypes are different from the BUB-1 PD^{mut}. Hence, our study highlights this important distinction between the two PLK-1 populations, and we hope our lab and/or others will be able to provide a mechanistic explanation in the near future.

Moreover, does CENP-C PD^{mut} affect CENP-C localization itself, or localization of KNL-1? Mapping which kinetochore components get hyper-recruited with CENP-C PD^{mut} (testing components from centromeric chromatin to the microtubule interface) would help delineate at which step CENP-C bound PLK-1 acts in regulating kinetochore assembly.

We have obtained new data showing that HCP-4 itself is not hyper-recruited in the CENP-C PD^{mut}. We have now included these data as New Figure 1E.

For other kinetochore components, we intend to provide a more mechanistic explanation of our phenotypes in future work. We are not able to exhaustively test all kinetochore components at this stage, but we are generating several strains we need to be able to get the full picture in the future.

Fig 3: Phosphorylation of KBP-2 by PLK-1 is clearly shown- were any phospho-sites identified that regulate its interaction with CENP-C? Or was there any mass spec analysis conducted of the phosphorylated proteins? While a detailed mechanism is not necessary for publication, any connection between potential PLK-1-mediated phosphorylation (e.g. of CENP-C itself or of the MIS-12 complex) and the observed limiting of outer kinetochore protein recruitment would greatly elevate the impact of the work.

We have now included new data for in vitro phospho-site identification of CENP-C, the KBP-2:MIS-12 dimer, and KNL-3 (New Table S1). While making a connection between PLK-1-mediated phosphorylation and kinetochore recruitment would be very tempting, this would be highly speculative without structural data. We considered using the AlphaFold2 structure of the MIS-12 complex bound to CENP-C; however, because we could still not i) produce a stable MIS-12 complex* and ii) the KBP-2:MIS-12 dimer does not bind CENP-C in vitro, we are not sufficiently confident on the prediction. We continue to work actively on reconstituting the MIS-12 complex and its binding to CENP-C.

Minor Points:

How efficient/effective is the feeding RNAi for CENP-C or BUB-1? A control showing knockdown of the endogenous protein (either imaging of an endogenously tagged strain in the presence of RNAi or a western blot showing depletion of the endogenous protein) is needed to confirm that the RNAi worked.

For CENP-C depletion, we observed the published depletion phenotype (Oegema et al, 2001): no mitotic chromosome segregation and aberrant chromosome organisation (See Figure 1B). Additionally, we are providing here an representative image of the GFP::CENP-C signal being lost in *cenp-c(RNAi)* oocytes (See below).

We have removed unpublished data that had been provided for the referees in confidence.

For BUB-1, we have shown BUB-1 depletion efficiency in our previous manuscript (Taylor et al. 2023) and we monitor for mitotic (delayed anaphase onset) and meiotic (defective chromosome alignment and segregation) phenotypes in every experiment. We have added below a comparison between a wild type and a *bub-1(RNAi)* oocytes, both expressing BUB-1::GFP.

© 2024. Published by The Company of Biologists under the terms of the Creative Commons Attribution License (https://creativecommons.org/licenses/by/4.0/).

We have removed unpublished data that had been provided for the referees in confidence.

Line 156: Typo, “to focus or further analysis” -> “to focus our further analysis”

Thanks for noting this error, which has now been corrected.

Line 163: Possible incomplete sentence

Thanks for noting this error, which has now been corrected.

Line 211: Typo, “yeloow” to “yellow”

Thanks for noting this error, which has now been corrected.

Line 230: Figure 2 legend for 2E is incorrect (chromosome distance, not centrosome to centrosome distance assay) and legend for 2F is missing (centrosome separation assay).

Thanks for noting this error, which has now been corrected.

Figure 1B: Images are grainy/pixelated, looks like an import issue. Please upload some clearer images.

This must have been a problem related to PDF conversion. We are now providing the original files for every Figure, where the quality is high.

Figure 2: n for 2D-F are missing, please show. Also the fluorophores for that experiment aren’t labeled.

We have now added the n and the fluorescent signal source.

Figure 3B: n is missing, please show.

N and n have been added to the graph, wich is now Figure 3C.

Fig 4D- would be nice to have images of PLK-1 alongside the quantification.

As requested, we have now added representative images of the PLK-1 kinetochore and centrosome levels in New Figure 4D and moved the quantification to panel E.

S1A: Decrease in PLK-1 with IF in the CENP-C PD^{mut} looks much stronger than the in vivo imaging-are the IF images quantifiable? Reason for difference between IF and in vivo imaging?

We do not know the reason for this discrepancy. There is an inherent degree of variability in both methodologies, more so in immunofluorescence. This is the main reason why we decided to base our quantifications on the live samples as this is a more controlled set-up. While IFs are not quantitative, we did add the image in supplementary as the main message still stands: PLK-1 levels decrease in both cases. We are adding below an example where PLK-1 is still detected in the CENP-C PD^{mut} (although clearly less than wild type).

We have removed unpublished data that had been provided for the referees in confidence.

S1B- would be nice to have BUB-1 quantification (the images don’t look appreciably different but would be nice to have quantification to show that they’re the same).

We added the quantification for BUB and this is now presented in the New Figure S1C.

S2B would be nice to have quantification for NDC80 recruitment (just at metaphase would be fine, just to go with the images showing increase with the CENP-C mutant)

© 2024. Published by The Company of Biologists under the terms of the Creative Commons Attribution License (https://creativecommons.org/licenses/by/4.0/).

NDC-80 quantifications have now been added for mitosis (New Figure S2B) and for meiosis (New Figure S2D).

Strain table: There appear to be strains missing- I don't see the strain with both HCP-4 PD^{mut}.

This as well as other missing strains were added to the strain list.

REVIEWER 2

Major points:

The authors show in Figure 2E that chromosomes separate prematurely in the CENP-C PD mutant; however, this phenotype is not apparent in Figure 1D. How do the authors explain this difference?

We thank the reviewer for raising this. Since the timing effect has not been introduced at this point in the manuscript, we matched the images by mitotic 'stage' rather than time. We have now made sure to indicate this in the text and in the figure legend. Additionally, we have also added labelling within the figure to indicate this. This applies to New Figures 1B, 1F, and 2A. After introduction of the timing effect (Figure 2C), time stamps are shown.

Combining mutations of the Polo-docking sites of BUB-1 and HCP-4/CENP-C abrogates PLK1 recruitment to the kinetochores during meiosis, causing major chromosome segregation defects. Therefore, how do the authors exclude the possibility that the defects they see during mitosis are not a consequence of the meiotic defects? They should at least discuss that point.

We agree with the reviewer, and we have added some text on this issue. Importantly, we observe the mitotic PLK-1 reduction in the small number of embryos with two polar bodies (i.e. the ~25% of embryos where the two segregations steps have occurred, as reported in our previous manuscript – Taylor et al, eLife 2023). We would also like to point out that as opposed to meiosis, there appears to be some PLK-1 remaining in the double BUB-1/CENP-C PD mutants (Figure 4D,E), which might explain the difference in sensitivities between meiosis and mitosis. We are currently investigating whether there are other mitotic PLK-1 receptors.

The authors should better characterize the phenotype of the CENP-C PD mutant by monitoring other kinetochore components (MIS12 subunits and others). How specific is the phenotype? Is only MIS12 deregulated?

While we have not conducted an exhaustive screen of kinetochore components, the 'first' defect we noted going from the centromere to the outer kinetochore is at the level of the MIS-12 complex. We also show that the NDC-80 complex is deregulated in the CENP-C PD mutant. For other kinetochore components, we intend to provide a more mechanistic explanation of our phenotypes in future work. We are not able to exhaustively test all kinetochore components at this stage, but we are generating several strains we need to be able to get the full picture in the future.

As for the phenotypic analysis, not all embryos have the same length, so the authors should normalize the centrosome-to-centrosome and chromosome distance to embryo length (Figures 2D and 2F).

We have now measured the length of wild type and CENP-C PD^{mut} embryos in different genetic backgrounds and found no significant differences, ruling out changes in embryo length as the cause of the phenotypes described. This is shown in the New Figure 2G.

Most of Figure 2B, where the authors show that the HCP-4 mutant does not impact CDC-20, could be moved to the supplementary material.

While we appreciate the reviewer's view, we believe this 'negative' result should remain as a main figure because it provides a strong indication that CENP-C-bound PLK-1 does not regulate a key BUB-1-bound PLK-1 target.

© 2024. Published by The Company of Biologists under the terms of the Creative Commons Attribution License (https://creativecommons.org/licenses/by/4.0/).

The authors show that PLK-1 phosphorylates the MIS12 subunit in vitro, but this potentially interesting observation has not been followed up, leaving the reader with little more information.

We have now included new data we have obtained for in vitro phospho-site identification of CENP-C, the KBP-2:MIS-12 dimer, and KNL-3 (New Table S1). While making a connection between PLK-1-mediated phosphorylation and kinetochore recruitment would be very tempting, this would be highly speculative without structural data. We considered using the AlphaFold2 structure of the MIS-12 complex bound to CENP-C; however, because we could still not i) produce a stable MIS-12 complex and ii) the KBP-2:MIS-12 dimer does not bind CENP-C in vitro, we are not sufficiently confident on the prediction. We continue to work actively on reconstituting the MIS-12 complex and its binding to CENP-C.

Minor points:

Some sentences still need to be finished, and some words must be included! lane 86, "To answer this question, we monitored?"

Thanks to the reviewer for noting this, which has now been corrected.

The drawing in Figure 1A needs to be more accurate. During anaphase, the mitotic spindle is asymmetrically positioned, resulting in a larger anterior and a smaller posterior cell. Embryos in positions 5, 6, and 7 should be turned to 180°C.

We thank the reviewer for highlighting this. We have decided to remove the last two drawings as we do not have data with 2-cell embryos and therefore these were not needed. We have also corrected the anaphase drawings to account for the asymmetry.

The drawing in Figure S2A would be helpful in the main Figures for showing the HCP-4/CENP-C position at the kinetochore.

We have now added the drawing as New Figure 3A.

The authors should be consistent with the nomenclature. Sometimes, they use the *C. elegans* nomenclature and, at other places, the human one. It is confusing. They could present the human and *C. elegans* nomenclature in a short table at the beginning or the double nomenclature throughout the manuscript, as done for KNL-3^{DSN1}. Generally speaking, it is preferable to use the *C. elegans* nomenclature to talk about the proteins of this organism.

We have corrected the sentence according to the suggestion.

Lane 120: add we imaged the endogenously tagged "MIS12 subunit" KNL-3/DSN1 subunit.

We have corrected the sentence according to the suggestion.

Figure 2F needs to be described in the figure legend.

We have made sure all panels are described in the figure legends.

Add timings in Panels 1B, 1D, 2A, 2D, 2E, 3A, S1B

Some figures are based on mitotic stage and others on timing. We have now added information on the stage/timing to all panels and the corresponding figure legends.

The number of experiments conducted (N) and embryos analyzed (n) is not always clearly indicated.

Information on the number of experiments and embryos analysed has now been added.

The overall quality of some figures could be improved (e.g., Fig 1B, 1D).

© 2024. Published by The Company of Biologists under the terms of the Creative Commons Attribution License (https://creativecommons.org/licenses/by/4.0/).

We believe the issue with quality must have been the pdf conversion and made sure all the original files we have submitted are of the highest quality.

Scale bars are missing Figure 4C

We have added the scale bar to Figure 4C.

REVIEWER 3

Specific points:

Abstract: Most proteins are referred to with the mammalian nomenclature in the abstract, with no dash (i.e. PLK1 instead of PLK-1). However, there are instances where PLK-1 and CDC-20 are written with dashes. Make the nomenclature consistent (or be sure to only use the dash when you are referring to the worm homolog).

We thanks the reviewer for highlighting this issue and we have now made the appropriate chages in the nomenclatrure.

Line 55: it is stated that there is a reduction in PLK-1 levels after CENP-C depletion, but from the figure it looks like this reduction is only in the chromosomal levels (PLK-1 appears to be at similar levels at the centrosomes). Modify the sentence to change the phrase to "reduction in chromosomal PLK-1 levels" for clarity.

The reviewer is correct and we have changed the sentence for clarity.

Line 60-61: In this manuscript the CENP-C point mutant is described as "CENP-C PDmut" , whereas in the previous paper where this mutant was first described, it was referred to as "hcp-4 T163A". It is fine to use a different notation in this manuscript but I think it would be easier for the reader to compare and synthesize the findings from the two manuscripts if, the first time this mutant is mentioned, you define it the way you did previously (e.g. "…a CENP-C point mutant that disrupts its polo docking motif (hcp-4 T163A, hereafter referred to as CENP-C PDmut)"…)

This is a useful suggestion and we have changed the sentence.

The schematic in Figure 2E does not show any interpolar microtubules in the spindle midzone. I suggest using the same spindle diagram as in Figure 1A, for consistency.

We changed the schematic in Figure 2E to match that of the New Figure 1A. We thank the reviewer for helping us maintain consistency throughout the manuscript.

Although this would already be clear to kinetochore a:icionados, I would edit the sentence on line 120 to make it clear that KNL-3DSN1 is a component of the Mis12 complex (novice readers may not know this and may be confused as to why you are looking at this protein).

We added this information.

Although most of the conclusions in the manuscript are well quantified, Figure 3D, S1A, S1B, S2B, and S2C show only a single image for each condition. The authors should provide some sort of quantification to better support these findings. At a minimum, the authors should state in the figure legend how many oocytes were analyzed and looked like the representative image (e.g. "x/x oocytes imaged had increased KNL-3/NDC-80 intensity in the mutant…").

We have added all the appropriate quantifications and information on sample sizes.

Typos:
- Line 10: should be "the nuclear envelope"
- Line 67: "Figure D" should be "Figure 1D"
- Line 129: delete "in"
- Line 211: yellow is misspelled

© 2024. Published by The Company of Biologists under the terms of the Creative Commons Attribution License (https://creativecommons.org/licenses/by/4.0/).

- Line 86: I think you are missing a word at the end of this line – add “assessed”?
- Line 96: “lead” should be “led”
- Line 156: “or” should be “our”
- Line 161: refers to “Figure 4X” – this should be “4D”
- Line 162: I think the word “that” should be deleted?
- Line 182: “in” should be “on”

We thank the reviewer for noting all these typos, which we have now corrected.

Line 101: a brown arrow is mentioned but it looks red to me on the figure. May just be an issue with my screen, but consider changing the color of the arrow on the figure.

We darkened the shade of brown to make the schematic clearer.

Second decision letter

MS ID#: JOCES/2024/262327

MS TITLE: CENP-C-targeted PLK-1 regulates kinetochore function in *C. elegans* embryos

AUTHORS: Laura Bel Borja, Samuel JP Taylor, Flavie Soubigou, and Federico Pelisch

I am happy to tell you that your manuscript has been accepted for publication in Journal of Cell Science, pending standard publication integrity checks. You will find referee reports on this version and a few requests are made to improve clarity or consistency. Please take these into consideration before submitting a final version of your manuscript.

Reviewer 2

Advance summary and potential significance to field

Here, the authors use the C. elegans embryo to dissect the mechanisms and function of PLK-1 recruitment to the inner kinetochore. They showed that abrogating PLK-1 recruitment to the kinetochores by combining point mutations on the Polo-docking sites of HCP-4/CENP-C and BUB-1 causes severe chromosome segregation defects.

Comments for the author

My apologies to the authors for the delay in sending back my review. While the manuscript has improved it requires additional corrections to ensure its quality and readability before publication.
1) The nomenclature is still non-consistent throughout the manuscript, which makes the text hard to read and comprehend for a naïve reader. Make the nomenclature consistent
Lane 38 PLK1/PLK-1
Lane 42, 43 “the sole CCAN component CENP-CHCP-4
Lane 51: We have recently identified HCP-4, the C. elegans orthologue of CENP-C
Lane 56 : cenp-c(RNAi)
Lane 59 : hcp-4T163A

2) The sentences inserted between lines 57 and 64 need more clarity. They disrupt the flow and make the transition difficult to understand. The authors should consider revising this part starting with this introductory sentence, which sets the problem:
“Since CENP-C is required for kinetochore assembly and consequent BUB-1 kinetochore recruitment lower chromosomal PLK-1 levels could be the trivial consequence of depleting BUB-1 from the kinetochore. We, therefore etc…..

3) To the comment “Add timings in Panels 1B, 1D, 2A, 2D, 2E, 3A, S1B,” the authors responded, “Some figures are based on mitotic stage and others on timing. We have now added

© 2024. Published by The Company of Biologists under the terms of the Creative Commons Attribution License (https://creativecommons.org/licenses/by/4.0/).

information on the stage/timing to all panels and the corresponding figure legends.” The reviewer does not understand this response. In Figure 1G, the authors show a graph where they quantified the levels of PLK-1::sGFP on chromatin throughout mitosis in wild-type versus CENP-C (PD mutant) relative to NEBD time 0. Therefore they should be able to add the timing to the pics presented in panels B and F. It would help the reader to link the images to the graph.

4) Typo Lane 270 “Combined Abrogation

Reviewer 3

Advance summary and potential significance to field

This manuscript by Borja et.al. reveals functions for inner kinetochore PLK-1 in C. elegans mitosis, separate from outer-kinetochore-targeted PLK-1. Overall, I found these findings to be interesting and of value to the cell division field as they reveal new insights into mechanisms driving the mitotic divisions.

Comments for the author

The authors have done a good job addressing reviewer concerns. Related to my own comments, the text modifications they made have improved the clarity of the manuscript, and the added quantifications have improved its rigor. In addition the authors performed new experiments in response to other reviewer concerns that have further strengthened the manuscript.

In my read-through of the manuscript, I found a few minor errors that should be fixed prior to publication (these are minor issues that were introduced when the authors added the new data, that should be straightforward to fix).

- In figure 1C, three embryos are shown. I am assuming that these represent three different stages of mitosis in wild-type embryos (prometaphase, metaphase anaphase), but since there aren’t labels on the rows, or information in the figure legend, this is not clear. Labels in the figure would help clarity, and the figure legend should note that these images are of wild-type embryos and that multiple stages of mitosis are shown. Also, I couldn’t find a callout to this panel in the results section – this should be added.

- Paragraph on lines 54-64. The wording of this paragraph was a little confusing – when new data were added to this part of the manuscript in the revision, it broke the flow of the story (because you transitioned from saying that you depleted CENP-C using RNAi to then talking about the PD mutant, before you explained why that mutant was useful). I would rearrange something like: “We used endogenously sfGFP-tagged PLK-1 to study its kinetochore recruitment and found a clear reduction in chromosomal PLK-1 levels after CENP-C depletion (‘cenp-c(RNAi)’) at all mitosis stages (Figure 1B). Since CENP-C is required for kinetochore assembly and consequent BUB-1 kinetochore recruitment, lower chromosomal PLK-1 levels could be the trivial consequence of depleting BUB-1 from the kinetochore. We therefore used a CENP-C mutant that cannot target PLK-1 but where CENP-C is still present on chromatin. In this mutant, Threonine 163 within the polo docking (PD) motif in CENP-C is mutated to Alanine so it can’t be phosphorylated (hcp-4T163A, hereafter referred to as ‘CENP-C PDmut’) (Figure 1D). Threonine 163 phosphorylation was detected in fixed embryos using a phospho-specific antibody and, as expected this signal was lost in the PD mutant (Figure 1 C,E).”
- For the kinase assays in Figure 3G, is there a reason that CDK1 and Aurora B are denoted with mammalian nomenclature rather than C. elegans (i.e. CDK-1 and AIR-2)?
I am assuming that you must have used mammalian proteins for these assays, but I couldn’t find this information in either the figure legends or materials and methods. Assuming I didn’t just miss this information, it should be added to the manuscript to make it clear how you obtained these kinase (purchased? Purified yourself?) and which species they are from.

Typos:
- Line 16: BUB11 should be BUB-1
- Line 45: “division” should be “divisions”

© 2024. Published by The Company of Biologists under the terms of the Creative Commons Attribution License (https://creativecommons.org/licenses/by/4.0/).

- Line 140: Drosophila should be in italics
- Line 270: should be “abrogation”
- Line 416: should “of the KNL-3” be “of the KNL-3:KBP-1 dimer”? Or “of KNL-3”? Fix somehow.
- Line 425: “of the CENP-C” should be “of CENP-C”

© 2024. Published by The Company of Biologists under the terms of the Creative Commons Attribution License (https://creativecommons.org/licenses/by/4.0/).
